# Asymmetric Hybrid Polyoxometalates: A Platform for Multifunctional Redox‐Active Nanomaterials

**DOI:** 10.1002/anie.201912046

**Published:** 2019-10-24

**Authors:** Elizabeth Hampson, Jamie M. Cameron, Sharad Amin, Joungman Kyo, Julie A. Watts, Hiroki Oshio, Graham N. Newton

**Affiliations:** ^1^ GSK Carbon Neutral Laboratory for Sustainable Chemistry University of Nottingham Nottingham NG7 2GA UK; ^2^ Graduate School of Pure and Applied Sciences University of Tsukuba Tennodai 1-1-1 Tsukuba 305-8571 Japan; ^3^ Nanoscale and Microscale Research Centre University of Nottingham Nottingham NG7 2RD UK; ^4^ State Key Laboratory of Fine Chemicals Dalian University of Technology 2 Linggong Rd. Dalian 116024 P. R. China

**Keywords:** clusters, hybrid materials, polyoxometalates, redox properties, supramolecular chemistry

## Abstract

Access to asymmetrically functionalized polyoxometalates is a grand challenge as it could lead to new molecular nanomaterials with multiple or modular functionality. Now, a simple one‐pot synthetic approach to the isolation of an asymmetrically functionalized organic–inorganic hybrid Wells–Dawson polyoxometalate in good yield is presented. The cluster bears two organophosphonate moieties with contrasting physical properties: a chelating metal‐binding group, and a long aliphatic chain that facilitates solvent‐dependent self‐assembly into soft nanostructures. The orthogonal properties of the modular system are effectively demonstrated by controlled assembly of POM‐based redox‐active nanoparticles. This simple, high‐yielding synthetic method is a promising new approach to the preparation of multi‐functional hybrid metal oxide clusters, supermolecular systems, and soft‐nanomaterials.

## Graphical abstract

**A one‐pot approach** to the isolation of an asymmetrically functionalized organic–inorganic hybrid Wells–Dawson polyoxometalate is presented. The cluster bears two organophosphonate moieties with contrasting physical properties: a chelating metal‐binding group and a long aliphatic chain unit that facilitates solvent‐dependent self‐assembly into soft redox‐active nanostructures.

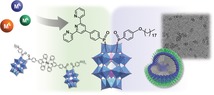

Polyoxometalates (POMs) are anionic nanoscale metal oxide clusters comprising early transition metals in their highest oxidation states. They continue to attract significant interest owing to their vast structural diversity, excellent stability, and versatile chemical properties, making them ideal building blocks for functional materials.^1^, ^2^, ^3^ Their capacity to undergo reversible multi‐electron redox processes is of particular interest, and renders them promising photo/electrocatalysts in a variety of systems.^4^, ^5^

The covalent integration of organic moieties and POMs is an exciting and rapidly expanding area within the field.^6^ Through the design of the individual components the intrinsic properties of the resulting hybrid systems can be fine‐tuned. This allows hybrid POMs to exhibit diverse functionality^7^, ^8^, ^9^ and facilitates their assembly into a range of supramolecular nanostructures for use in devices or catalytic systems.^10^, ^11^, ^12^, ^13^, ^14^, ^15^, ^16^, ^17^ The development of simple synthetic procedures that maximize control in the tailoring of multi‐functionality in hybrid systems is an ongoing challenge. Almost all reported bi‐functionalized hybrid POMs are symmetric systems, in which two identical organic groups are tethered to the inorganic core. The controlled addition of two different organic groups to create an asymmetric hybrid system would open up far greater opportunities for fine control over the function and physicochemical properties, allowing them to be tailored towards highly specific or advanced applications. A few key examples of asymmetrically functionalized POM hybrids have been reported but remain rare owing to the significant challenge posed by their synthesis and purification.^18^ For instance, Cronin et al. recently demonstrated the use of HPLC to isolate a precursor asymmetric Mn‐Anderson hybrid in which one ligand terminates in a protecting group.^19^ Notably, however, this requires demanding, expensive, and time‐consuming methods (such as chromatography or slow fractional crystallization) followed by multiple synthetic steps to obtain the asymmetric product.^20^, ^21^, ^22^ Reported examples of asymmetric hybrid POMs that exhibit complex functionalities are even fewer,^21^, ^23^ and (with the notable exception of a series of elegant though hydrolytically unstable organotin hybrid POMs reported by Lacôte, Thorimbert, and Hasenknopf)^24^ are limited to the Mn‐Anderson cluster type, which displays limited photo‐ and redox‐activity compared to many POMs. Conversely, advanced functionalization strategies targeting clusters such as the Wells–Dawson anion, with its rich redox activity and highly tunable organic hybrid structures,^25^, ^26^, ^27^ will provide new pathways for the isolation of advanced, multi‐functional soft‐nanostructures^28^, ^29^ and metal‐directed extended assemblies.^30^, ^31^ Ultimately, this could allow for the design of materials with switchable morphologies and functions in a range of different media, leading to new applications in catalysis, drug‐delivery systems, or encapsulated nano‐reactors.^32^, ^33^

Herein, we present a simple, inexpensive, and high‐yielding synthetic approach to isolate the first reported example of an asymmetric bifunctional Wells–Dawson hybrid POM, obtained by exploiting the different solubilities of three hybrid POMs produced in the crude mixture. The cluster bears a chelating metal‐binding group, and a long aliphatic chain unit that allows solvent‐dependent self‐assembly into soft nanostructures (Scheme 1). The simple nature of this approach (requiring no specialist lab equipment) should allow its application in the development of a whole new class of asymmetric hybrid POMs.

**Scheme 1:**
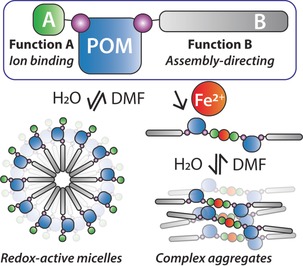
An asymmetric hybrid POM bearing two different organic moieties (A and B) and its tunable solvent and cation‐dependent self‐assembly.

Functionalization of polyoxotungstates with aryl phosphonate groups enhances their photochemical properties and can be employed to direct their self‐assembly into micellar superstructures.^29^, ^34^, ^35^, ^36^ Here, we aimed to introduce two distinct, orthogonal functionalities via asymmetric functionalization and designed two suitable aryl phosphonates accordingly: (PO_3_C_21_H_16_N_3_) (TPY), a terpyridine‐based ligand, and (PO_4_C_24_H_43_) (C_18_), an aliphatic chain group (see the Supporting Information). Hybridization was then conducted based on the previously reported method.^34^ In a one‐pot acid‐catalyzed condensation reaction, one molar equivalent each of TPY and C_18_ were reacted with one molar equivalent of the potassium salt of the mono‐lacunary Dawson‐type anion, [P_2_W_17_O_61_]^10−^ (see the Supporting Information). A 1:1 solvent mixture of *N*,*N*′‐dimethylformamide (DMF) and acetonitrile was used to adequately solubilize both ligands. After reaction completion, ^31^P NMR spectroscopy indicated the presence of three species (Figure 1; Supporting Information, Figure S1), later fully characterized as a racemic mixture of the two enantiomers of the asymmetric hybrid, K_4_(C_2_H_8_N)_2_[P_2_W_17_O_57_(PO_3_C_21_H_14_N_3_)(PO_4_C_24_H_41_)] (**1**) and the symmetric hybrids, (C_2_H_8_N)_6_[P_2_W_17_O_57_(PO_3_C_21_H_14_N_3_)_2_] (**2**), and K_2_(C_2_H_8_N)_4_[P_2_W_17_O_57_(PO_4_C_24_H_41_)_2_] (**3**). In the ^31^P NMR spectrum of **1** the signals for the covalently‐bound ligands, TPY and C_18_, appear at 13.31 and 16.77 ppm respectively, while the corresponding peaks appear at 14.10 and 15.96 ppm in the spectra of the respective symmetric structures, **2** and **3**. Isolation of the desired product, **1**, was achieved as follows. A large excess of ether was added to the reaction mixture upon which the solution turned cloudy. On centrifugation, a mixture of orange‐brown and green solid was collected and the yellow filtrate decanted. Removing the solvent from the filtrate leaves an orange‐brown crystalline solid that by ^31^P NMR is identified as **3**. The remaining crude solid, a mixture of **2** and **1**, was re‐dissolved in acetonitrile, in which **2** is sparingly soluble and can be separated by centrifugation. Finally, an excess of ether was used to re‐precipitate **1** while leaving any traces of **3** in solution. The process of dissolution in minimum acetonitrile to remove any insoluble **2**, followed by re‐precipitating **1** from the filtrate with ether, can be repeated as necessary until no signals corresponding to **2** or **3** are visible by ^31^P or ^1^H NMR (Figure 1). **1** can be reliably isolated in good yield (typically 10–25 % total yield) and excellent purity.

**Figure 1:**
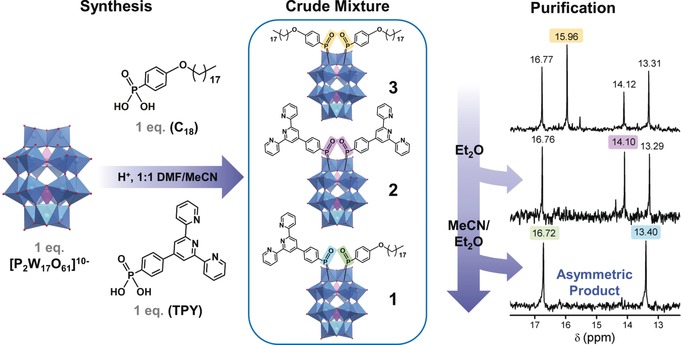
Synthesis and purification of the asymmetric hybrid‐POM, **1**, and the symmetric by‐products, **2** and **3**, illustrating the purification process. ^31^P NMR of the reaction mixture in DMSO‐D_6_ is shown on the right, taken after each purification step (top: crude; middle **1**+**2**; bottom: **1** only). Chemical shifts corresponding to each product are color‐coded as shown (note that for simplicity, only the positive chemical shift region for the organophosphonate ^31^P nuclei is presented). Blue polyhedra {WO_6_}, pink polyhedra {PO_4_}, red spheres oxygen. Cations and solvent molecules are omitted for clarity.

The composition and purity of **1** was confirmed by ^1^H NMR, ^31^P NMR, ESI‐MS, elemental (CHN) analysis, thermogravimetric (TGA) analysis, and FTIR (see the Supporting Information). ^1^H NMR confirms the presence of both the aliphatic carbon chain and the aromatic TPY group in a 1:1 stoichiometric ratio. Interestingly, slight shielding of the TPY phosphorus and deshielding of the C_18_ phosphorus relative to their ^31^P chemical shifts in the respective symmetric hybrids suggests a degree of electronic communication via the POM core of the asymmetric structure.^37^

Following successful isolation of **1**, we explored the unique multifunctionality of the hybrid POM system, as derived from its asymmetric structure. First, we examined the self‐assembly of **1** enabled by the aliphatic chain ligand, C_18_, which imparts amphiphilic character to the hybrid POM (the POM core and TPY group acting as a polar head‐group). **1** was first dissolved in acetonitrile and then 9 equivalents of water were added to facilitate spontaneous self‐assembly. Dynamic light scattering (DLS) experiments on a 1.4 mm solution confirmed the formation of nanoscale assemblies of low dispersity with a hydrodynamic diameter (*D*_h_) of approximately 6 nm (Supporting Information, Figure S18). In agreement with this, Cryo‐TEM analysis of a solution of **1** in water–acetonitrile (9:1 v/v) deposited and frozen on a graphene‐oxide and holey carbon‐supported Cu grid showed spherical structures with diameters of 4–7 nm (Supporting Information, Figure 2). Corresponding ^1^H and ^31^P NMR experiments showed broadening/disappearance of peaks in a D_2_O–CD_3_CN (9:1 v/v) mixture, indicative of reduced T_2_ transverse relaxation time arising from the slow tumbling of nanostructures (Supporting Information, Figure S21).

**Figure 2:**
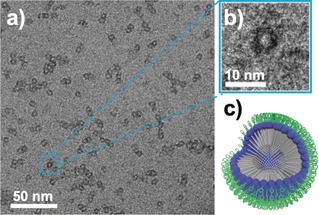
a) Cryo‐TEM imaging of micellar assemblies of **1** formed in 1.4 mm water–acetonitrile (9:1 v/v) solution; b) expanded view of a single micelle; c) proposed structure of the micellar assemblies of compound **1**. Blue spheres {P_2_W_17_} units, gray rods **C_18_** units, green rings **TPY** units.

Cyclic voltammetry (CV) of **1** in a 0.1 m DMF solution of TBA⋅PF_6_ revealed four distinct quasi‐reversible redox processes in the potential range of −0.5 to −2.25 V vs. Fc/Fc^+^ (Supporting Information, Figure S10), all of which are positively shifted relative to the parent Wells–Dawson anion, [P_2_W_18_O_62_]^6−^, as is typical of organophosphonate hybrid POMs.^37^ Furthermore, *E*_1/2_ potentials of **1** were found to be intermediate to those of the respective symmetric hybrids, **2** and **3**, with redox processes in **2** more positively shifted by an average of 5 mV and those of **3** more negative by an average of 20 mV, relative to the equivalent redox couples of **1** (see the Supporting Information for details).

Recently, we showed that self‐assembled micellar nanostructures formed from hybrid POM species exhibit different redox properties to their constituent molecular species.^29^, ^35^ CV experiments were conducted on **1** dissolved in a 0.1 m H_2_SO_4_:CH_3_CN (9:1, v/v) mixture, allowing for spontaneous assembly into micellar species as described above. Here, just two redox processes were observed between −0.5 and 0.5 V (vs. Ag/AgCl) in contrast to the three seen in pure DMF (Supporting Information, Figure S15), as appears typical of micellar species of this type.^29^, ^35^ Notably, characteristic molecular redox behavior can be rapidly recovered by addition of DMF to the electrolyte solution, indicating the dynamic nature of this system and offering interesting potential for the development of responsive or switchable electrochemical devices. This behavior also corresponds closely to that seen for our previously reported symmetric surfactant hybrid system,^35^ and for the symmetric product **3** (Supporting Information, Figure S21).

Post‐functionalization of **1** was investigated by reacting the hybrid‐POM with 0.5 molar equivalents of FeCl_2_. After stirring the reactants at RT overnight in acetonitrile, the product, K_7_(C_2_H_8_N)_3_{Fe[P_2_W_17_O_57_(PO_3_C_21_H_14_N_3_)(PO_4_C_24_H_41_)]_2_} **Fe‐1** (Figure 3 a), was collected by removal of the solvent. Mass spectrometry confirmed the formation of a POM‐Fe‐POM dimeric structure, and all aromatic peaks associated with the TPY groups were shifted in the ^1^H NMR, indicating the presence of a single new species. Peak shifts of both organophosphoryl signals (C_18_ downfield; TPY upfield) in the ^31^P NMR spectrum of **Fe‐1** demonstrate a degree of electronic conjugation across the whole asymmetric system. The dark purple color of the product is characteristic of a low spin d^6^ Fe^2+^ center, and UV/Vis absorption spectroscopy confirms the presence of a peak at 576 nm corresponding to a Fe^2+^‐TPY metal–ligand charge‐transfer (MLCT) band (Supporting Information, Figure S13). CV studies in DMF reveal a reversible Fe^2+/3+^ redox couple (*E*_1/2_=0.543 V vs. Fc/Fc^+^) alongside the 4 quasi‐reversible POM‐centered processes (Supporting Information, Figure S16).

**Figure 3:**
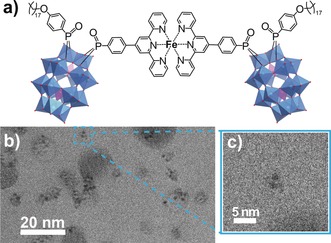
a) Molecular structure of the dimeric hybrid‐POM complex **Fe‐1**. b) Cryo‐TEM imaging of assemblies of **Fe‐1** formed in 1.4 mm water‐acetonitrile (9:1 v/v) solution; c) expanded view of a single dimeric molecule.

The combination of metal coordination and self‐assembly of **1** was then investigated by dissolving **Fe‐1** in a water–acetonitrile (9:1 v/v) mixture at RT. DLS studies indicated the formation of nanoscale aggregates (Supporting Information, Figure S20) with *D*_h(avg)_=4 nm, and NMR studies again showed the disappearance of the peaks in D_2_O‐CD_3_CN (9:1 v/v) solution (Supporting Information, Figure S22). CryoTEM analysis confirmed the presence of the assemblies (Figure 3 b), but unlike the corresponding studies on **1**, the aggregates were of variable shape and were not hollow. This is reasonable, given that **Fe‐1** lacks the head/tail structure of **1**, and is therefore unlikely to form typical micellar assemblies. Indeed, the assemblies appear to resemble those recently reported by Izzet and co‐workers, who demonstrated the organic solvent and transition metal‐dependent assembly of symmetric terpyridine‐functionalized POMs into a range of nanoscale architectures.^30^, ^38^ It is also apparent that many of the POMs (dark areas on the micrograph) appear in pairs (Figure 3 c), consistent with the expected molecular structure of **Fe‐1**, in which the POM lobes should be separated by approximately 2 nm. As with **1**, the assemblies exhibited contrasting electrochemical behavior to their molecular state, which was recovered upon addition of DMF (Supporting Information, Figure S17).

In summary, we have isolated the first stable asymmetric Wells–Dawson hybrid POM using a simple and inexpensive procedure for the synthesis of novel multi‐functional hybrid materials based on molecular metal oxides. We have explored the multifunctionality of these asymmetric assemblies, driven by the nature of the appended organophosphoryl groups. The orthogonal and complementary functions of the aliphatic chain (facilitating solvent‐dependent self‐assembly) and the chelating group (allowing transition metal binding and also influencing supramolecular interactions) allow us to tune the performance of the hybrid system through careful post‐functionalization and to obtain unique redox‐active soft nanostructures. This work provides a robust, modular, and easily accessible strategy for the creative design and development of new multi‐functional hybrid materials, with a range of potentially novel applications as redox‐, photo‐, and catalytically active soft nanomaterials.

## Conflict of interest

The authors declare no conflict of interest.

## Supporting information

As a service to our authors and readers, this journal provides supporting information supplied by the authors. Such materials are peer reviewed and may be re‐organized for online delivery, but are not copy‐edited or typeset. Technical support issues arising from supporting information (other than missing files) should be addressed to the authors.

Supplementary
