# Supplementary material for: Asymmetric Hybrid Polyoxometalates: A Platform for Multifunctional Redox‐Active Nanomaterials
Source: Angew Chem Int Ed Engl. 2019 Oct 24;58(50):18281–5. doi: 10.1002/anie.201912046 (PMC6916258; doi:10.1002/anie.201912046)
Supplement: Supplementary file 1 — Supplementary [file ANIE-58-18281-s001.pdf]

Angewandte Chemie
Eine Zeitschrift der Gesellschaft Deutscher Chemiker

Supporting Information

Asymmetric Hybrid Polyoxometalates: A Platform for Multifunctional Redox-Active Nanomaterials

*Elizabeth Hampson, Jamie M. Cameron, Sharad Amin, Joungman Kyo, Julie A. Watts, Hiroki Oshio, and Graham N. Newton**

anie_201912046_sm_miscellaneous_information.pdf

Table of Contents

Methods 1
Syntheses and Materials 2
NMR Characterisation of 1, Fe-1, 2 & 3 8
Mass Spectrometry of 1 & Fe-1 12
FTIR Analysis 14
UV/Vis Absorption Spectroscopy 15
Electrochemistry of 1 & Fe-1 15
DLS & NMR characterisation of assemblies of 1 & Fe-1 18
Electrochemistry of 2, 3, and {P_2W_{18}}, and DLS of 3 21
References 23

Methods

^{1}H NMR, ^{13}C NMR and ^{31}P NMR-spectra were obtained using Bruker Ascend^{TM} 400MHz and 500MHz spectrometers.

Infra-red spectra were measured using a Bruker Alpha FTIR spectrometer with a platinum ATR module.

UV/Vis absorption spectroscopy was performed on a Cary 5000 UV/VIS NIR spectrophotometer.

Electrospray ionisation mass spectrometry (ESI-MS) was performed on Bruker MicroTOF and Impact II spectrometers.

CHN microanalysis was carried out using an Exeter Analytical CE-440 Elemental Analyser.

Thermogravimetric analysis (TGA) was carried out using a TA Instruments Discovery TGA. Samples were heated from 25-1000°C under air at 10°C/min.

Dynamic Light Scattering (DLS) measurements were acquired using a Malvern Instrument Nano-ZS Zetasizer at room temperature.

Cryo-TEM samples were prepared and imaged using a Gatan CP3 cryoplunge and a JEOL 2100 Plus operating at 200 kV. In detail, the sample was deposited (3 µL) onto a graphene oxide / holey carbon copper grid, held in tweezers (25 °C, 80% humidity), and blotted (1.5 s), before plunging into liquid ethane (-172 °C) to vitrify. The sample was maintained under liquid

nitrogen (-196 °C) during transfer (Gatan 926 cryo sample holder) to the TEM, with the temperature held around –176 °C throughout imaging with a Gatan (Smartset model 900) cold stage controller. Images were recorded (Gatan Ultrascan 100XP camera), with a nominal underfocus value of 3-5 µm and a 60 µm objective aperture to enhance phase contrast.

Electrochemical measurements were performed on a CHI600e (CH Instruments) workstation. Cyclic voltammetry (CV) experiments under non-aqueous conditions were performed using a three-electrode arrangement; working electrode (glassy carbon, d= 3mm), reference electrode (Ag wire) and a counter electrode (Pt wire). Experiments were performed in $TBA.PF_6$ (0.1 M) as the supporting electrolyte in dry DMF. All potentials with ferrocene as an internal standard, are quoted relative to the $E_{1/2}$ of the ferrocene redox couple. Experiments under aqueous conditions were performed using a three-electrode arrangement; working electrode (glassy carbon, d= 3mm), reference electrode (Ag/AgCl) and a counter electrode (Pt wire). All solutions were purged with argon for 10 mins and kept under a positive pressure of Ar for the duration of the experiment. All measurements were performed at a scan rate of 100 mVs^{-1}.

Syntheses and Materials

All reagents were obtained from commercial sources and were used without further purification.

Synthesis of K_{10}-[$P_2W_{17}O_{61}$] {P_2W_{17}}

K_{10}-[$P_2W_{17}O_{61}$] and its precursor, K_6-[$P_2W_{18}O_{62}$], were synthesised according to procedures in literature.[1, 2]

Synthesis of (**TPY**)

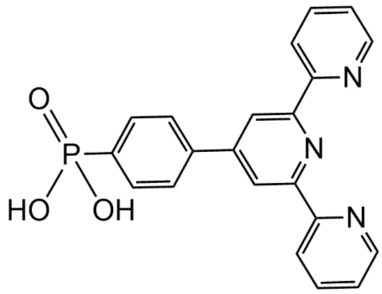

Synthesis of BrPhTPY - $C_{21}H_{14}N_3Br$

4-bromobenzaldehyde (5 g, 27.0 mmol), 2-acetylpyridine (6.06 mL, 54 mmol) and KOH (4.54 g, 81 mmol) were dissolved in ethanol (300 mL). Ammonium hydroxide solution (35 %, 62 mL) was added whilst stirring, and the mixture was heated at reflux (75 °C) for 24h. The mixture was filtered and the collected precipitate was washed with ethanol and allowed to dry in air. The crude product was purified by re-precipitation from hot chloroform using cold ethanol, to yield BrPhTPY as a white powder. Further product was obtained via a second crop from the filtrate and these were combined and dried under vacuum (2.71 g, 26 %).

^{1}H NMR (400.1 MHz, $CDCl_3$): δ= 8.73 (ddd, *J*=4.8, 1.8, 0.9 Hz, 2H; Ar-H), 8.70 (s, br, 2H; Ar-H), 8.67 (dt, *J*=7.9, 1.1 Hz, 2H; Ar-H), 7.91-7.87 (td, *J*=7.5, 1.8, 6H; Ar-H), 7.79-7.77 (m, 2H; Ar-H), 7.65-7.63 (m, 2H; Ar-H), 7.38-7.35 (ddd, *J*=7.5, 4.8, 1.2 Hz, 2H; Ar-H) ppm; **MS (ESI)** *m/z* $[M+H]^+$ calcd for $[C_{21}H_{14}N_3Br_1+H]^+$: 390.0426, found: 390.0426.

Synthesis of $PO(OEt)_2$PhTPY - $C_{25}H_{24}N_3PO_3$

Ethanol (80 mL), diethylphosphite (3.32 mL, 25.8 mmol) and triethylamine (1.5 mL, 10.8 mmol) were added to BrPhTPY (2.5 g, 6.4 mmol), $Pd(OAc)_2$ (145 mg, 0.65 mmol) and PPh_3 (507 mg, 1.9 mmol) under argon and the mixture was heated at reflux (75 °C) whilst stirring for 22h. The solution was allowed to cool to RT before filtering, and the solvent was removed in vacuo. The yellow precipitate was recrystallised from hot CH_3CN twice to yield $PO(OEt)_2$PhTPY as an off-white crystalline solid (2.16 g, 74 %).

^{1}H NMR (400.1 MHz, $CDCl_3$) δ= 8.75 (s, br, 2H; Ar-H), 8.74-8.72 (ddd, *J*=4.8, 1.8, 0.9 Hz, 2H; Ar-H), 8.69-8.67 (dt, *J*=7.9, 1.1 Hz, 2H, Ar-H), 8.01-7.93 (m, 4H; Ar-H), 7.91-7.87 (td, *J*=7.7, 1.8 Hz, 2H; Ar-H), 7.35-7.38 (ddd, *J*=7.5, 4.8, 1.2 Hz, 2H; Ar-H), 4.24-4.08 (m, 4H; CH_2), 1.35 (t, *J*=7.0 Hz, 6H; CH_3) ppm**;** **^{31}P NMR** (162 MHz, $CDCl_3$) δ= 18.31 ppm; **MS (ESI)** *m/z* $[M+H]^+$ calcd for $[C_{25}H_{24}N_3O_3P_1+H]^+$: 446.1628, found: 446.1624.

Synthesis of (**TPY**), $PO(OH)_2$PhTPY - $C_{21}H_{16}N_3PO_3$

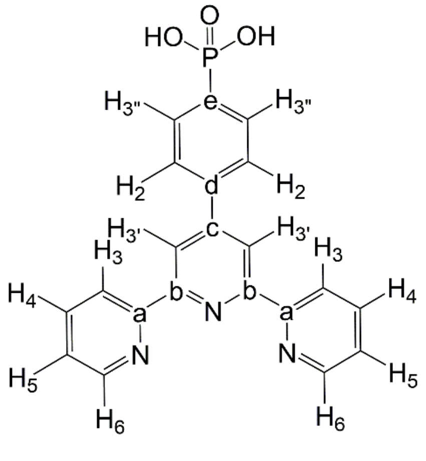

$PO(OEt)_2$PhTPY (2 g, 4.4 mmol) was dissolved in dry DCM (50 mL) and bromotrimethylsilane (1.8 mL, 13 mmol) was added under argon before the mixture was stirred at RT for 16h. The solvent was removed in vacuo, before adding a methanol-DCM mixture (40 mL, 1:1 v/v), and the solution was stirred at RT for 3h. The cloudy purple mixture was filtered to yield the crude product as a lilac powder. The crude was washed with hot CH_3CN and then ether, to give $PO(OH)_2$PhTPY as an off-white powder (1.15 g, 67 %).

^{1}**H NMR** (400.1 MHz, DMSO-d$_6$): δ 8.80-8.79 (ddd, *J*=4.8, 1.8, 0.9 Hz, 2H; $H_{6'}$), 8.77 (s, br, 2H; $H_{3'}$), 8.74-8.72 (dt, *J*=8.0, 1.1 Hz 2H; H_3), 8.12-8.08 (td, *J*=7.7, 1.8 Hz, 2H; H_4), 8.07-8.04 (m, 2H; H_2), 7.92-7.87 (m, 2H; $H_{3''}$), 7.60-7.57 (ddd, *J*=7.5, 4.8, 1.2 Hz, 2H; H_5) ppm; ^{13}**C NMR** (100.6 MHz, DMSO-d$_6$): δ 155.9 (s, C-b), 154.8 (C-a), 149.5 (C-6), 149.4 (C-e), 140.0 (C-c), 138.6 (C-4), 134.9 (C-d), 132.1/132.0 (C-2), 127.3/127.1 (C-3”), 125.3 (s, C-5), 121.8 (C-3), 118.8 (C-3’) ppm; ^{31}**P NMR** (162 MHz, DMSO-d$_6$): δ 11.95 ppm; **IR** (ATR): 665, 781, 894, 1071, 1133, 1241, 1288, 1393, 1424, 1527, 1595 cm^{-1}; **UV-Vis** (DMF): λ_{max} (ε)= 280 (41900), 315 (8750) nm (mol^{-1}Lcm^{-1}); **MS (ESI)** *m/z* [*M*+H]$^+$ calcd for [$C_{21}H_{16}N_3O_3P_1$+H]$^+$: 390.1002, found: 390.1003.

Synthesis of (**C$_{18}$**)

C$_{18}$ was synthesised as reported in our previous work. [3]

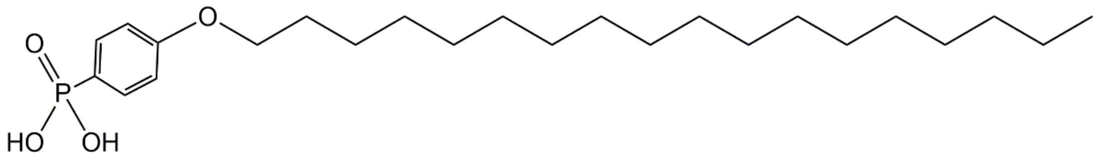

Synthesis of BrPhOC$_{18}$ – 1-bromo-4-(octadecyloxy)benzene - $C_{24}H_{41}BrO$

1-Bromooctadecane (2.85 g, 8.54 mmol) and 4-bromophenol (1.82 g, 10.5 mmol) were dissolved in DMF (40 mL). To this solution, KI (0.25 g, 1.51 mmol) and K_2CO_3 (4.51 g, 32.6 mmol) were added, and the reaction mixture was heated (80°C) for 20h. The reaction was then cooled to RT and the solvent was then removed *in vacuo*. The solid product was dissolved in diethyl ether (100 mL) and washed with 2M NaOH (2 x 100 mL), then brine (100 mL). The organic fraction was dried over $MgSO_4$ and the solvent was removed *in vacuo* to leave an orange oil. The crude product was then recrystallised from the minimal amount of hot ethanol to obtain **BrPhOC$_{18}$** as a white solid (3.07 g, 85 %).

^{1}**H NMR** (400 MHz, $CDCl_3$) δ= 7.43-7.32 (m, 2H; CH), 6.85-6.74 (m, 2H; CH), 3.93 (t, *J*=6.6 Hz, 2H; CH_2), 1.78 (dq, *J*=8.1, 6.6 Hz, 2H; CH_2), 1.46 (dq, *J*=11.7, 6.8 Hz, 2H; CH_2), 1.28 (s, 28H; CH_2), 0.97-0.85 (m, 3H; CH_3) ppm.

Synthesis of PO(OEt)$_2$PhOC$_{18}$ – diethyl (4-(octadecyloxy)phenyl)phosphonate - $C_{28}H_{51}O_4P$

To a microwave vessel, triethylphosphite (1.8 mL, 10.5 mmol) and anhydrous $NiCl_2$ (0.045 g, 0.35 mmol) was added, stirred and purged with argon for 10 mins. Dried BrPhOC$_{18}$ (1.49 g, 3.5 mmol) was added to the black mixture, heated (to dissolve) and purged with argon for a further 10 mins. The vessel was then heated in the microwave at 200 °C for 45 mins. The reaction mixture was then cooled to RT and dissolved in diethyl ether (100 mL). The solution was then filtered and centrifuged for 15 mins (8000 rpm). The black precipitate was filtered off and the solvent was evaporated *in vacuo* to leave a yellow oil. The crude product was then

dissolved in hot acetonitrile (60 mL) and filtered. Ethyl acetate (50 mL) was then added to the filtrate and the solvent was removed *in vacuo* to yield **$PO(OEt)_2PhOC_{18}$** as an off-white waxy solid (1.03 g, 61%).

^{1}H NMR (500 MHz, $CDCl_3$) δ 7.82–7.63 (m, 2H; CH), 7.04–6.89 (m, 2H; CH), 4.29–3.92 (m, 6H; CH_2), 1.81 (dt, *J*=14.0, 6.6 Hz, 2H; CH_2), 1.53–1.42 (m, 2H; CH_2), 1.40–1.17 (m, 34H; CH_2), 0.90 (t, *J*=6.9 Hz, 3H; CH_3) ppm; **^{31}P NMR** (202 MHz, $CDCl_3$) δ 19.7 ppm; **MS (ESI)** *m/z* $[M+H]^+$ calcd for $[C_{28}H_{51}O_4P+H]^+$: 483.36, found 483.36.

Synthesis of **(C_{18})**, $PO(OH)_2PhOC_{18}$ 4-(octadecyloxy)phenyl)phosphonic acid - $C_{24}H_{43}O_4P$

Dry DCM (6 mL) was added followed by bromotrimethylsilane (TMSBr) (1.95 mL, 14.78 mmol) to dried **$PO(OEt)_2PhOC_{18}$** (1.04 g, 2.07 mmol) under argon to form a yellow/green solution. The solution was then stirred for 20h at 30°C. Upon completion, the vessel was cooled to RT. The solvent and excess TMSBr was removed *in vacuo* to yield a yellow oil. To the flask, methanol-water (80:20 v/v, 30 mL) was added and stirred for 2 hours to form a white suspension. The mixture was then centrifuged (5 mins, 7000 rpm) and the solvent was decanted. The resulting solid was dried under vacuum to yield the crude as an off-white solid. The crude product was then washed with diethyl ether (2 x 25 mL), then stirred in hot acetonitrile (50 mL) and filtered, to yield **C_{18}** as a white powder (0.84 g, 96%).

^{1}H NMR (500 MHz, Methanol-*d_4*) δ 7.80-7.68 (m, 2H; CH), 7.01 (dq, *J*=9.3, 2.7 Hz, 2H; CH), 4.04 (t, *J*=6.5 Hz, 2H; CH_2), 1.89-1.74 (m, 2H; CH_2), 1.56-1.26 (m, 30H; CH_2), 0.92 (t, *J*=6.9 Hz, 3H; CH_3) ppm; **^{31}P NMR** (202 MHz, Methanol-*d_4*) δ 17.27 ppm; **MS (ESI)** *m/z* $[M-H]^-$ calcd for $[C_{24}H_{43}O_4P-H]^-$: 425.29, found: 425.29; **IR** (ATR): 449, 560, 542, 686, 826, 946, 1020, 1142, 1255, 1463, 1506, 1599, 2850, 2918 cm^{-1}; **Elemental Analysis** calcd (%) for $C_{24}H_{43}O_4P_1$: C 67.58, H 10.16, N 0, found: C 67.51, H 10.49, N 0.08.

Synthesis of asymmetric POM hybrid **(1),**

$K_4(C_2H_8N)_2[P_2W_{17}O_{57}(PO_3C_{21}H_{14}N_3)(PO_4C_{24}H_{41})].3H_2O$

K_{10}-$[P_2W_{17}O_{61}]$ (1.6 g, 0.35 mmol), **TPY** (0.14 g, 0.35 mmol), **C_{18}** (0.15g, 0.35 mmol), and KCl (0.52 g, 6.98 mmol) were suspended in DMF-CH_3CN (50 mL, 1:1 v/v) and stirred. 12M HCl (290 µL, 3.5 mmol) was added dropwise to the solution whilst stirring, and the mixture was heated to 85°C for 16h. The mixture was allowed to cool to RT before filtering. A large excess of ether was added (120 mL) to give a milky solution, which was centrifuged to give a dark orange-brown precipitate in a yellow filtrate. The solvent was decanted and the process of sonicating the precipitate in ether, centrifuging and decanting was repeated until the precipitate resembled a dry green-brown powder. The solid was sonicated in acetonitrile (10-15 mL) and centrifuged to separate an insoluble pale green-blue solid. The yellow filtrate was decanted and ether was added (25-30 mL) to it to precipitate a dark orange-brown solid, and leave a

pale yellow filtrate. The solid was collected by centrifugation, sonicated in ether and dried in air with gentle heating. The process of: re-dissolving in acetonitrile and centrifuging to remove any insoluble **2**, followed by re-precipitating **1** from the filtrate with ether, was repeated until no **2** or **3** was visible in the ^{31}P and ^{1}H NMR. The dry solid was finally dissolved in a minimum amount of acetone and centrifuged to separate any insoluble solid. The solvent was then removed in vacuo to yield **1** as a dark orange crystalline solid (390 mg, 22% (or 43%, assuming 50% is the max. recovery of asymmetric product statistically)).

^{1}H NMR (400.1 MHz, DMSO-d_6) δ= 8.95-8.88 (m, 6H; Ar-H), 8.33-8.29 (td, 2H; Ar-H), 8.33-8.09 (br, 4H; N**H₂**(CH₃)₂⁺) → $N\mathbf{H_2}(CH_3)_2^+$), 8.25-8.19 (m, 2H; Ar-H), 8.12-8.09 (m, 2H; Ar-H), 7.77-7.73 (m, 2H; Ar-H), 7.03-7.00 (dd, 2H; Ar-H), 4.04 (t, 2H; O-CH_2), 2.55 (t, 12H; $NH_2(C\mathbf{H_3})_2^+$), 1.74 (q, 2H; $C\mathbf{H_2}$-CH_3), 1.47-1.21 (m, 28H; -CH_2-), 0.86 (t, 3H; CH_3) ppm; **^{31}P NMR** (162 MHz, DMSO-d_6) δ= 16.72, 13.40, -11.32, -12.95 ppm; **IR** (ATR): 472 (s), 526 (s), 567 (s), 594, 718 (br, vs), 907 (s), 954 (s), 1084 (s), 1133 (w), 1253 (w), 1294 (w), 1387 (w), 1414 (w), 1463 (m), 1504 (w), 1531 (w), 1568-1595 (m), 1651 (w), 2850 (m), 2920 (m) cm^{-1}; **Elemental Analysis** calcd (%) for $K_4P_4W_{17}O_{64}C_{49}H_{71}N_5$: C 11.29, H 1.49, N 1.34, found: C 11.48, H 1.47, N 1.28; **TGA Analysis:** 1st step transition (30.6 to 64.3°C) wt loss= 1.200 % = loss of 3 H_2O (calcd= 1.050 %)**,** 2nd step transition (231.0 to 395.5°C) wt loss= 8.422 % = loss of 2 C_2H_8N (DMA) + C_{18} (cleaved at phosphonate linker unit) (calcd= 8.533 %).

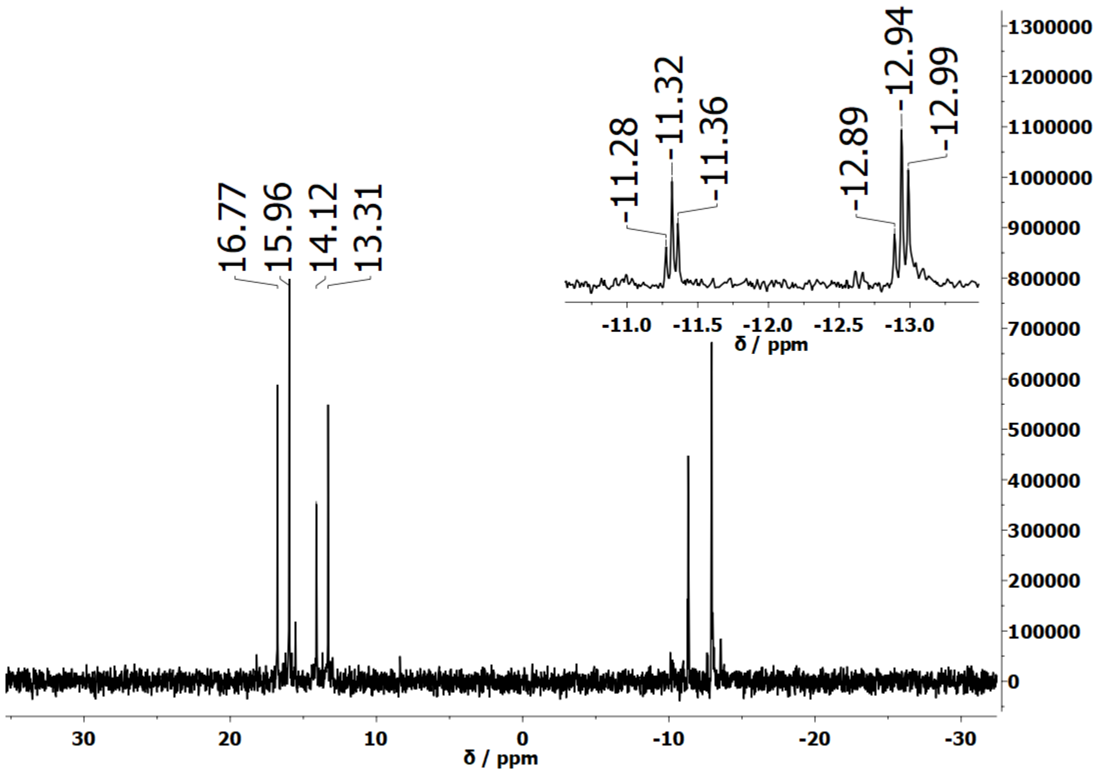

Figure S1. ^{31}P NMR of the crude mixture in the synthesis of **1** in DMSO-d_6. Inset shows an expansion of the peaks in the negative spectral region.

Synthesis of **Fe-1**, $K_7(C_2H_8N)_3[Fe(P_2W_{17}O_{57}(PO_3C_{21}H_{14}N_3)(PO_4C_{24}H_{41}))_2].2C_2H_8NCl.8H_2O$

$FeCl_2$ (2.47 mg, 0.0194 mmol) and **1** (200 mg, 0.0376 mmol) were dissolved in CH_3CN (5 mL) and stirred under argon at RT for 16h. The solvent was removed in vacuo to leave **Fe-1** as a dark purple solid that was washed with ether and dried in air with gentle heating (140 mg, 71%).

^{1}H NMR (500 MHz, DMSO-d$_6$) δ=9.78 (s, 4H), 9.16-9.05 (d, *J*=8.2 Hz, 4H; Ar-H), 8.78-8.64 (m, 4H; Ar-H), 8.44-8.34 (m, 4H; Ar-H), 8.31-8.14 (br, 6H; N**H$_2$**$(CH_3)_2^+$), 8.06-8.03 (m, 4H; Ar-H), 7.97-7.90 (m, 4H; Ar-H), 7.37-7.17 (m, 8H; Ar-H), 7.06-7.00 (m, 4H; Ar-H), 4.05 (t, 4H; O-C**H$_2$-**), 2.55 (t, 18H; NH_2(C**H$_3$**)$_2^+$), 1.79-1.72 (m, 4H; C**H$_2$**), 1.48-1.10 (m, 60H; -C**H$_2$**-), 0.86 (t, 6H; -C**H$_3$**) ppm; **^{31}P NMR** (202 MHz, DMSO-d$_6$) δ= 16.92, 13.15, -11.29, -12.92 ppm; **IR** (ATR): 473 (s), 523 (s), 566 (s), 718 (br, vs), 905 (s), 953 (s), 1050 (w), 1085 (s), 1136 (w), 1253 (w), 1295 (w), 1412 (w), 1428 (w), 1463 (m), 1504 (w), 1531 (w), 1568-1597 (m), 2848 (m), 2918 (m) cm^{-1}; **Elemental Analysis** calcd (%) for $K_7P_8W_{34}O_{136}C_{100}H_{166}N_{11}Cl_2Fe$: C 11.33, H 1.58, N 1.45, found: C 11.41, H 1.41, N 1.33; **TGA Analysis:** 1st step transition (37.9 to 66.1°C) wt. loss = 1.42 % = loss of 8 H_2O (calc'd = 1.36 %); 2nd step transition (258.7 1 to 443.9°C) wt. loss = 5.11 % = loss of 3 DMA + 2 DMA.Cl + C_{18} (cleaved at phosphonate linker unit) (calcd = 5.24 %).

Synthesis of symmetric POM hybrid **(2)**, $(C_2H_8N)_6[P_2W_{17}O_{57}(PO_3C_{21}H_{14}N_3)_2].3C_3H_7NO. 6H_2O$

The reaction conditions and relative quantities in the synthesis for **2** were as for **1**, except in 100% DMF and reacting 2 molar equivalents of **TPY** with K_{10}-$[P_2W_{17}O_{61}]$. An excess of ether was added to the filtrate to precipitate a dark blue-green oily layer. The mixture was centrifuged and then the solvent decanted. The oil was redissolved in the minimum volume of DMF and precipitated again with ether. Then, the process of centrifugation, decanting and sonication with ether was repeated until it resembled a dry powder. **2** was yielded as a green solid (1.9 g, 79%).

^{1}H NMR (400.1 MHz, DMSO-d$_6$,) δ= 8.81-8.79 (m, 4H; Ar-H), 8.78 (s, br, 4H; Ar-H), 8.71-8.68 (dt, 4H; Ar-H), 8.25-8.19 (m, 4H; Ar-H), 8.08-7.98 (m, 8H; Ar-H), 7.56-7.52 (ddd, *J*=7.5, 4.6, 4H; Ar-H) ppm; **^{31}P NMR** (162 MHz, DMSO-d$_6$,) δ= 14.20, -11.27, -12.90 ppm; **IR** (ATR): 426 (s), 523 (s), 575 (s), 718 (br, vs), 909 (s), 950 (s), 1022 (w), 1084 (s), 1133 (w), 1259 (w), 1300 (w), 1387 (w), 1414 (w), 1463 (m), 1531 (w), 1568-1595 (m), 2766 (br, m), 2961 (br, m) cm^{-1}; **TGA Analysis**: 1st step transition (39.0 to 72.7°C) wt loss= 1.891 % = loss of 6 H_2O (calcd= 1.972 %)**,** 2nd step transition (200.0 to 313.2°C) wt loss of 6.922 % + 3rd step transition (343.0 to 411.0°C) wt loss of 2.259 % = 9.181 % = 6 DMA + 3 DMF (calcd= 9.051 %).

Synthesis of symmetric POM hybrid **(3)**, $K_2(C_2H_8N)_4[P_2W_{17}O_{57}(PO_4C_{24}H_{41})_2].3C_3H_7NO .4H_2O$

The reaction conditions and relative quantities in the synthesis for **3** were as for **1**, reacting 2 molar equivalents of $\mathbf{C_{18}}$ with K_{10}-$[P_2W_{17}O_{61}]$. The solvent was removed *in vacuo* and the sticky solid was redissolved in acetone before it was left in the fridge (4-5°C) overnight. The solution was centrifuged and the yellow filtrate decanted from any precipitate. The solvent was then removed *in vacuo* to yield **3** as a crystalline orange solid (0.51 g, 69%).

^{1}H NMR (400.1 MHz, DMSO-d_6,) δ= 7.87 (dd, *J*=13.6, 8.5 Hz, 4H; Ar-H), 6.98 (dd, *J*=8.8, 3.3 Hz, 4H; Ar-H), 4.02 (t, *J*=6.5 Hz, 4H; O-CH_2-), 1.75-1.68 (m, 4H; -CH_2-), 1.45-1.18 (m, 60H; -CH_2-), 0.84 (t, *J*=6.9 Hz, 6H; -CH_3) ppm; **^{31}P NMR** (162 MHz, DMSO-d_6,) δ= 16.04, -11.38, -13.01 ppm; **IR** (ATR): 422 (s), 471 (s), 523 (s), 566 (s), 716 (br, vs), 906 (s), 955 (s), 1087 (s), 1136 (m), 1255 (w), 1292 (w), 1385 (w), 1412 (w), 1439 (w), 1463 (m), 1504 (m), 1595 (m), 1651 (s), 2850 (m), 2920 (m) cm^{-1}; **TGA Analysis**: 1st step transition (37.1 to 62.4°C) wt loss= 1.354 % = loss of 4 H_2O (calcd= 1.309 %)**,** 2nd step transition (280.7 to 312.7°C) wt loss of 13.557 % + 3rd step transition (319.6 to 417.9°C) wt loss of 6.856 % = 20.413 % = 4 DMA + 3 DMF + 2C_{18} (cleaved at phosphonate linker units) (calcd= 19.876 %).

NMR Characterisation of 1, Fe-1, 2 & 3

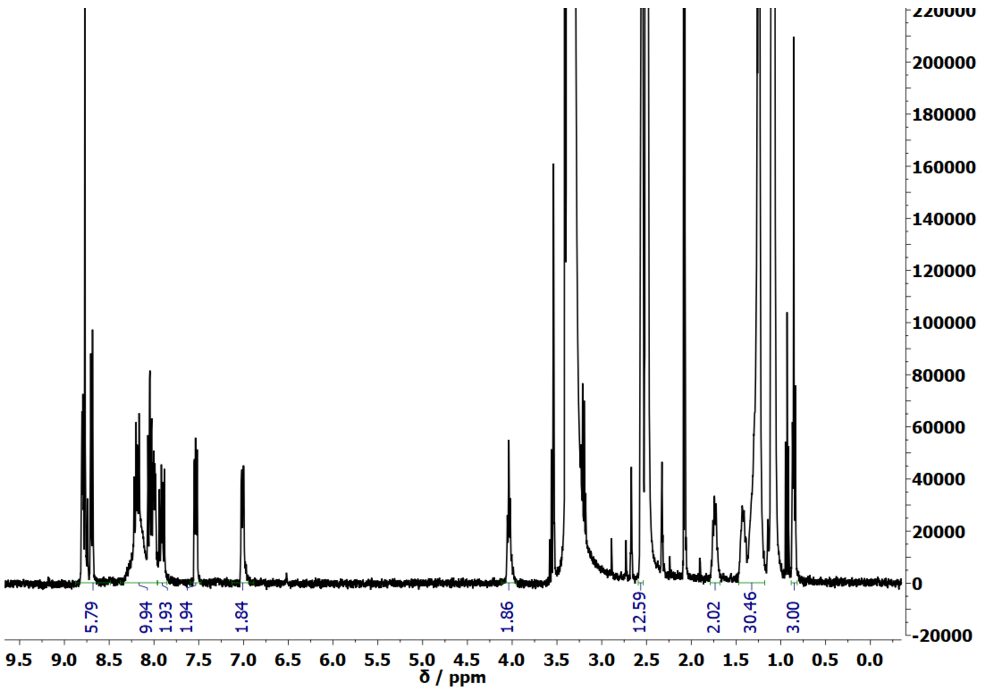

Figure S2. ^{1}H NMR of **1** in DMSO-d_6

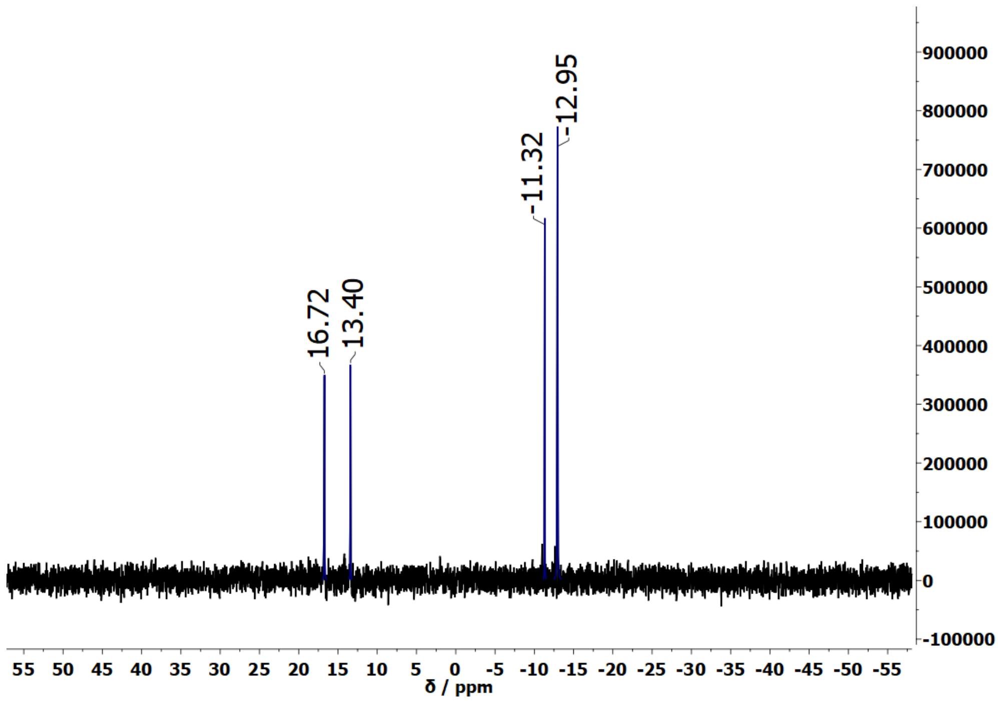

Figure S3. ^{31}P NMR of **1** in DMSO-d$_6$

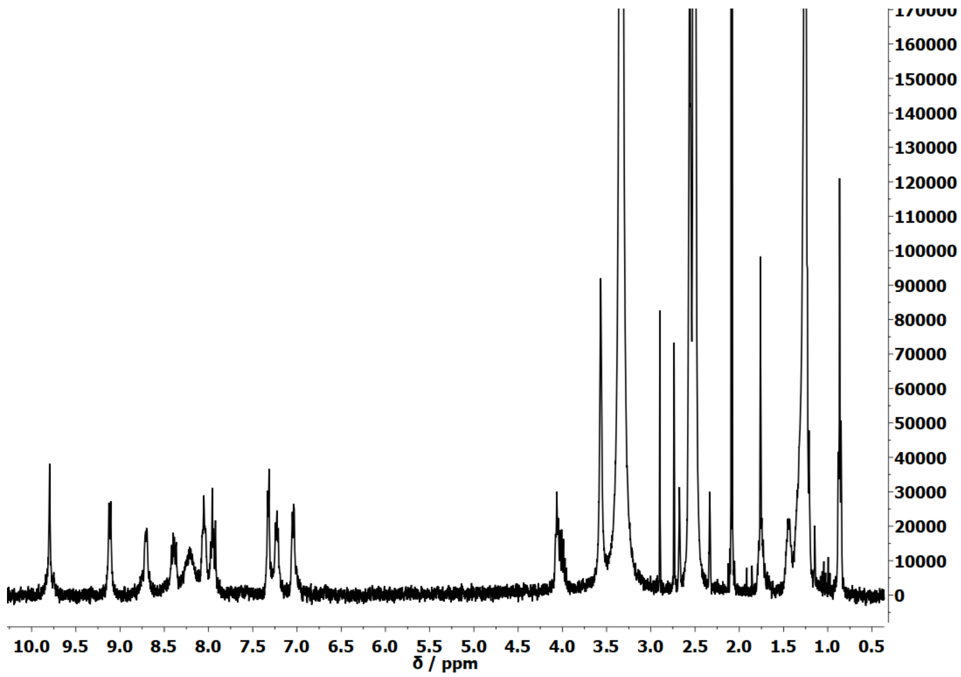

Figure S4. ^{1}H NMR of **Fe-1** in DMSO-d$_6$

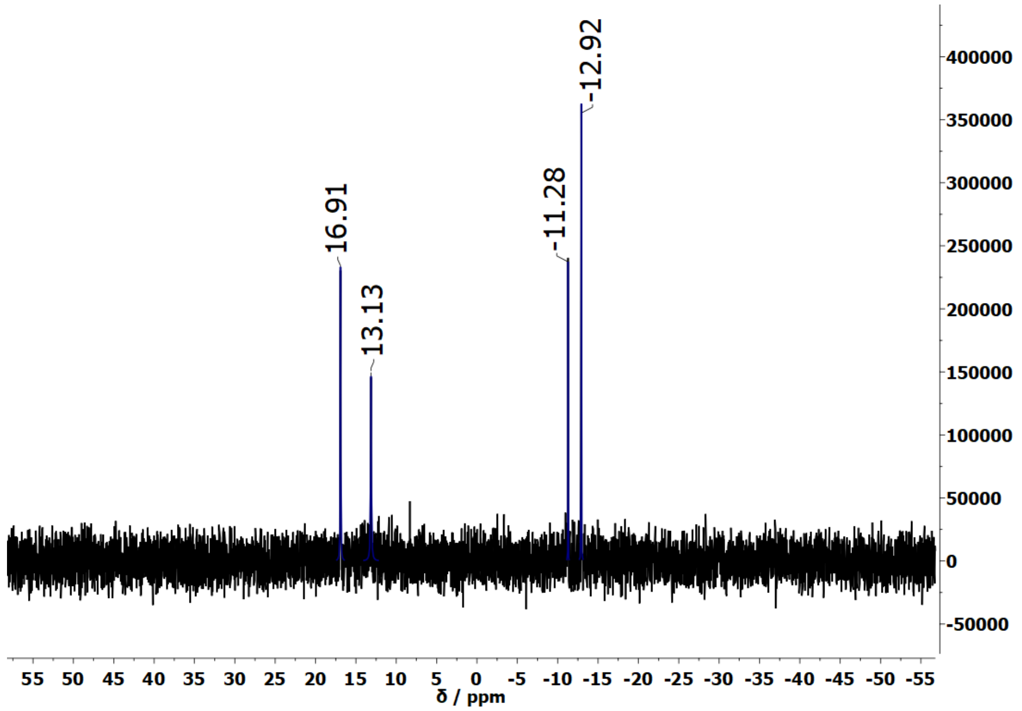

Figure S5. ^{31}P NMR of **Fe-1** in DMSO-d$_6$

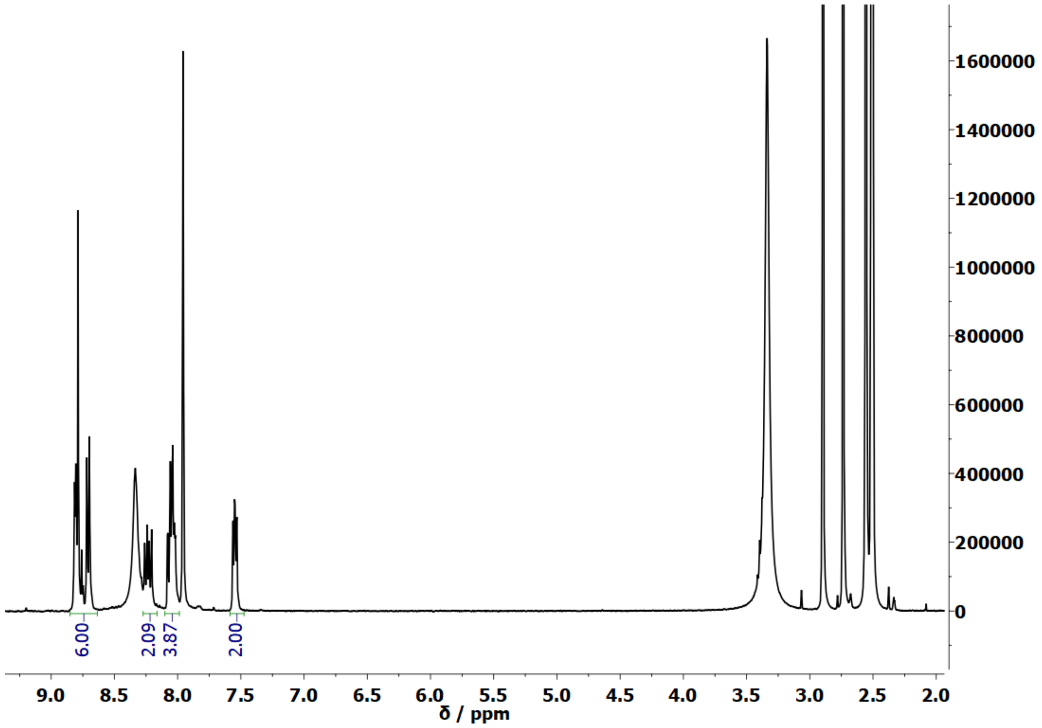

Figure S6. ^{1}H NMR of **2** in DMSO-d$_6$

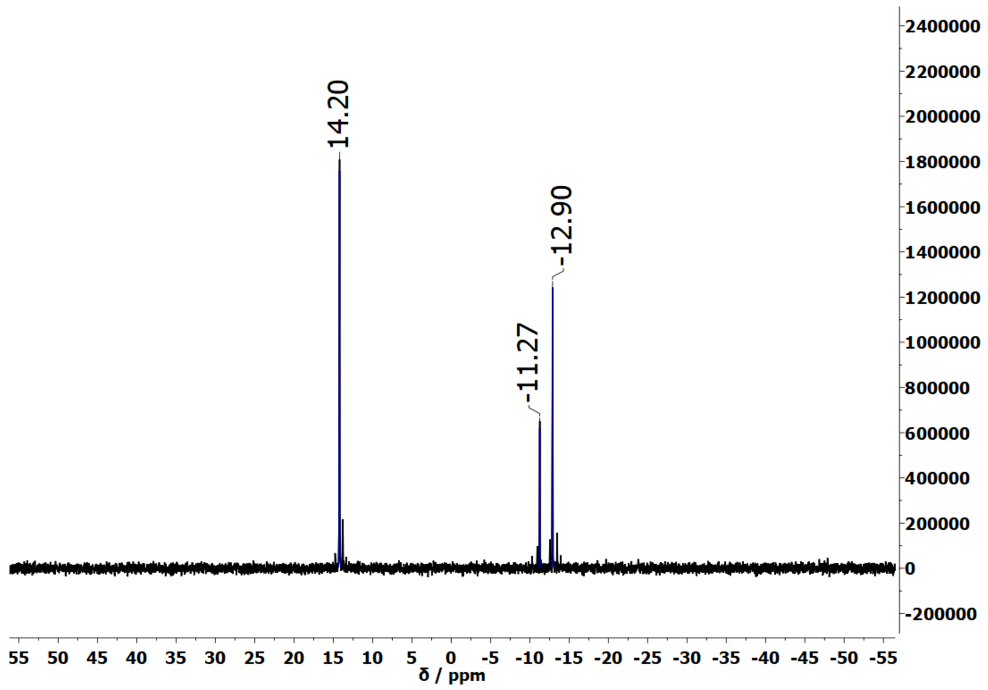

Figure S7. ^{31}P NMR of **2** in DMSO-d$_6$

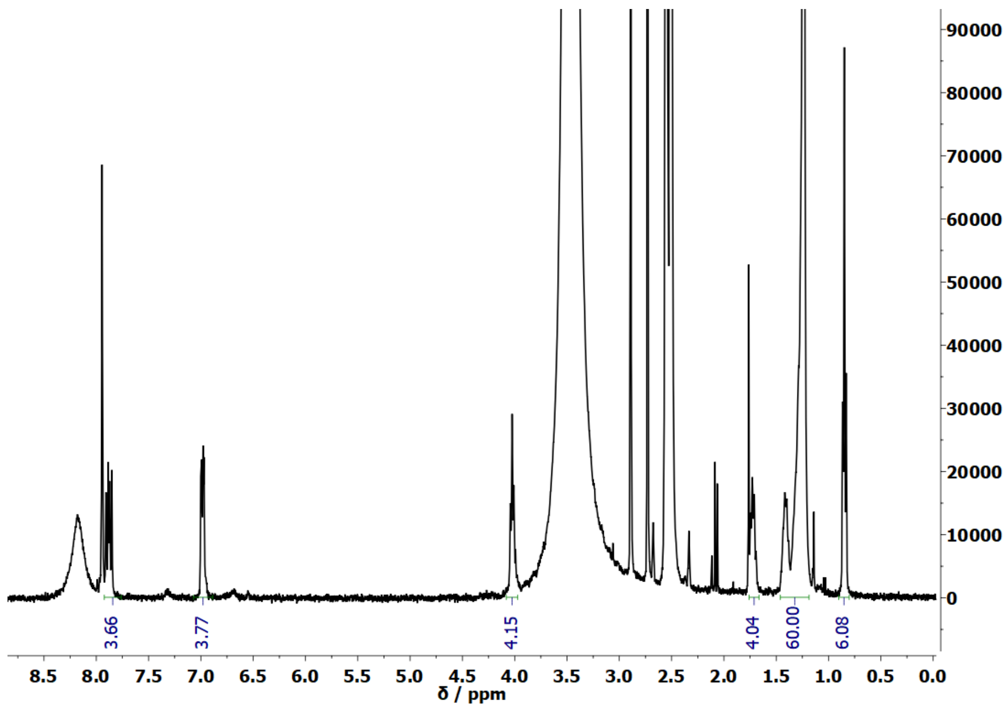

Figure S8. ^{1}H NMR of **3** in DMSO-d$_6$

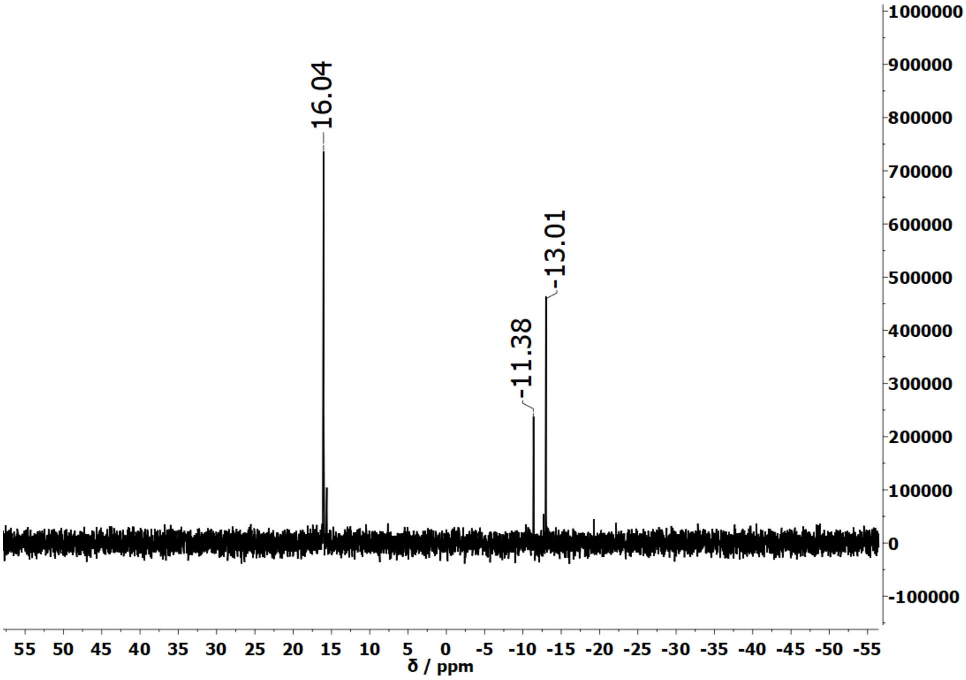

Figure S9. ^{31}P NMR of **3** in DMSO-d$_6$

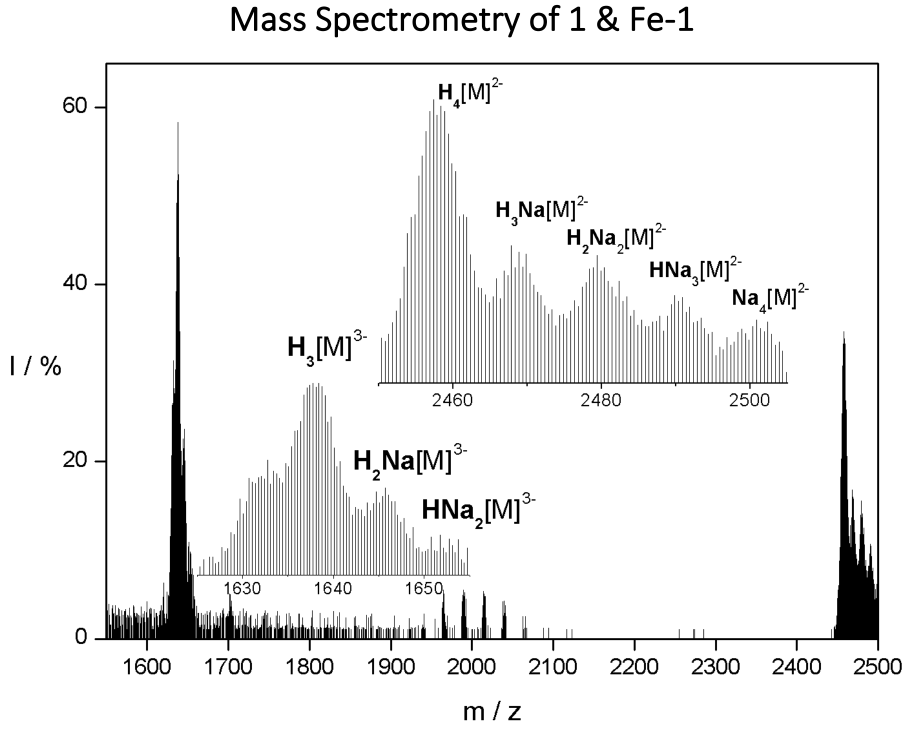

Figure S10. Negative mode ESI mass spectrum of **1** in acetonitrile. [M] = [$P_2W_{17}O_{61}(POC_{21}H_{14}N_3)(PO_2C_{24}H_{41})$]

Assignment	z	m/z (obs.)	m/z (calcd.)
**H_3**$[P_2W_{17}O_{61}(POC_{21}H_{14}N_3)(PO_2C_{24}H_{41})]$	3-	1638.0753	1638.0672
**H_2Na**$[P_2W_{17}O_{61}(POC_{21}H_{14}N_3)(PO_2C_{24}H_{41})]$	3-	1645.4042	1645.3945
**HNa_2**$[P_2W_{17}O_{61}(POC_{21}H_{14}N_3)(PO_2C_{24}H_{41})]$	3-	1652.7300	1652.7219
**H_4**$[P_2W_{17}O_{61}(POC_{21}H_{14}N_3)(PO_2C_{24}H_{41})]$	2-	2457.3475	2457.6050
**H_3Na**$[P_2W_{17}O_{61}(POC_{21}H_{14}N_3)(PO_2C_{24}H_{41})]$	2-	2468.3490	2468.5960
**H_2Na_2**$[P_2W_{17}O_{61}(POC_{21}H_{14}N_3)(PO_2C_{24}H_{41})]$	2-	2479.3527	2479.5870
**HNa_3**$[P_2W_{17}O_{61}(POC_{21}H_{14}N_3)(PO_2C_{24}H_{41})]$	2-	2490.3401	2490.5780
**Na_4**$[P_2W_{17}O_{61}(POC_{21}H_{14}N_3)(PO_2C_{24}H_{41})]$	2-	2501.3511	2501.5689

Table S1. M/z values observed and calculated for **1**

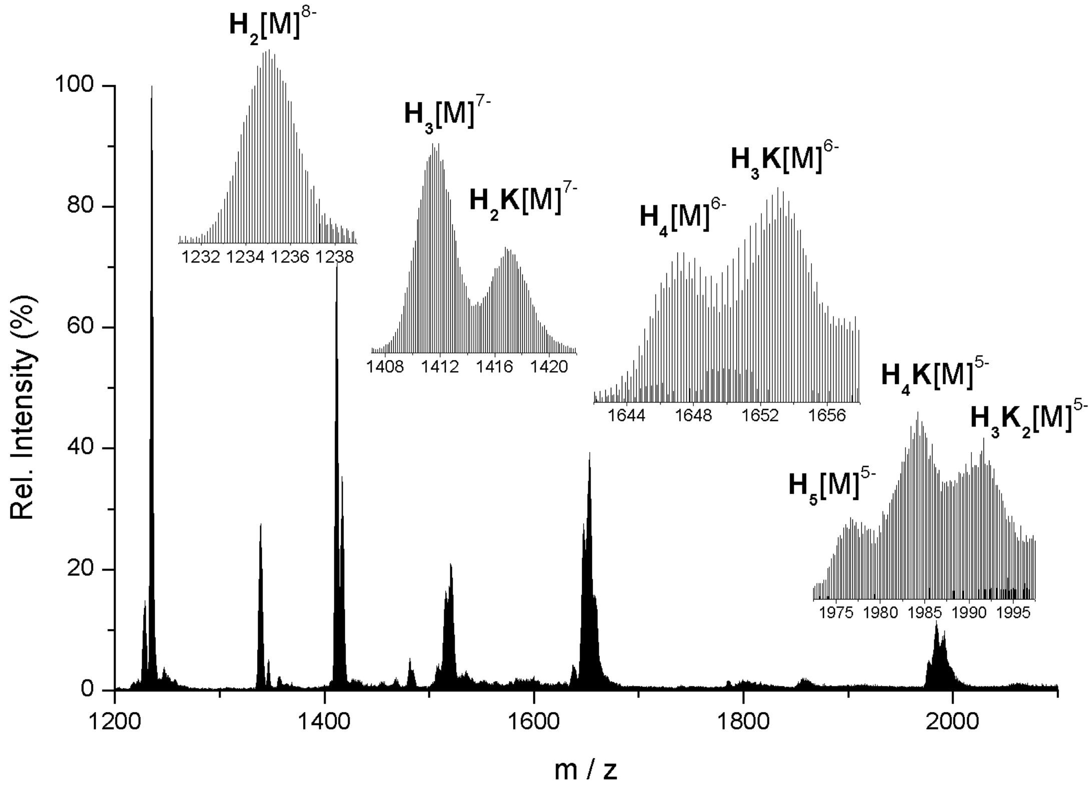

Figure S11. Negative mode ESI mass spectrum of **Fe-1** in acetonitrile. [M] = $[P_2W_{17}O_{61}(POC_{21}H_{14}N_3)(PO_2C_{24}H_{41})]_2$

Assignment	z	m/z (obs.)	m/z (calcd.)
**H_2Fe**$[P_2W_{17}O_{61}(POC_{21}H_{14}N_3)(PO_2C_{24}H_{41})]_2$	8-	1235.0435	1235.0383
**H_2Fe**$[P_2W_{17}O_{61}(POC_{21}H_{14}N_3)(PO_2C_{24}H_{41})]_2$	7-	1411.6222	1411.6164
**H_2KFe**$[P_2W_{17}O_{61}(POC_{21}H_{14}N_3)(PO_2C_{24}H_{41})]_2$	7-	1417.0442	1417.0386
**H_4Fe**$[P_2W_{17}O_{61}(POC_{21}H_{14}N_3)(PO_2C_{24}H_{41})]_2$	6-	1647.0585	1647.0539
**H_3KFe**$[P_2W_{17}O_{61}(POC_{21}H_{14}N_3)(PO_2C_{24}H_{41})]_2$	6-	1653.3864	1653.3798
**H_5Fe**$[P_2W_{17}O_{61}(POC_{21}H_{14}N_3)(PO_2C_{24}H_{41})]_2$	5-	1976.6718	1976.6663
**H_4KFe**$[P_2W_{17}O_{61}(POC_{21}H_{14}N_3)(PO_2C_{24}H_{41})]_2$	5-	1984.2644	1984.2574
**H_3K_2Fe**$[P_2W_{17}O_{61}(POC_{21}H_{14}N_3)(PO_2C_{24}H_{41})]_2$	5-	1991.8572	1991.8485

Table S2. M/z values observed and calculated for **Fe-1**

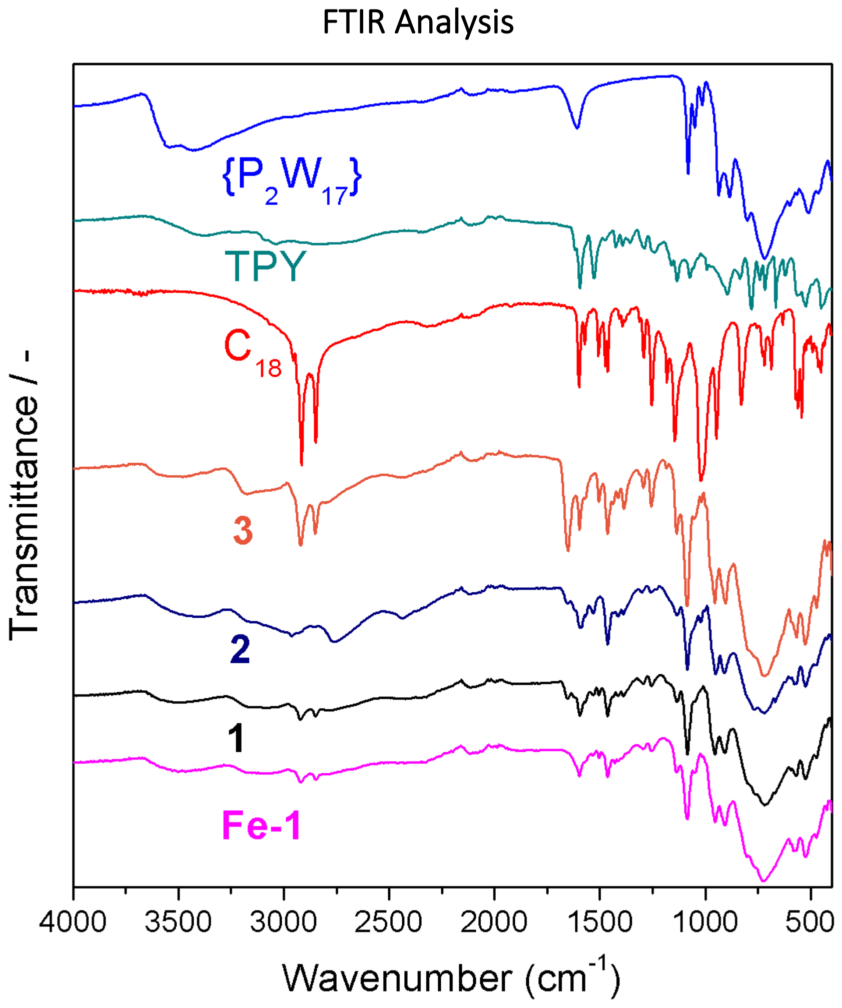

Figure S12. Comparison of ATR IR spectra of **1**, **Fe-1**, K_{10}-$[P_2W_{17}O_{61}]$ $\{P_2W_{17}\}$, ligands TPY & C_{18}, & symmetric hybrid POMs 2 & 3.

UV/Vis Absorption Spectroscopy

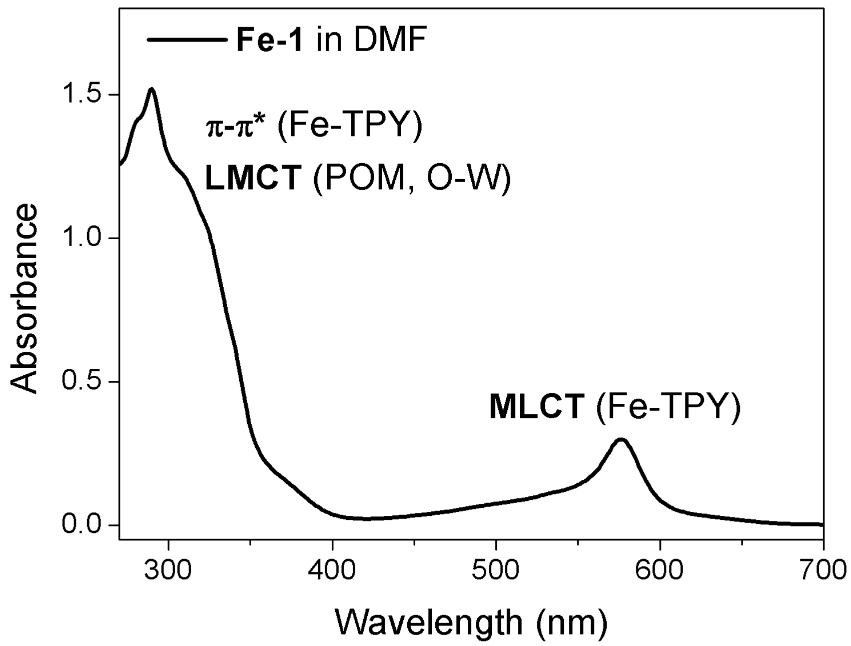

Figure S13. UV-Vis absorption spectrum of **Fe-1** in DMF (10 μM).

Electrochemistry of 1 & Fe-1

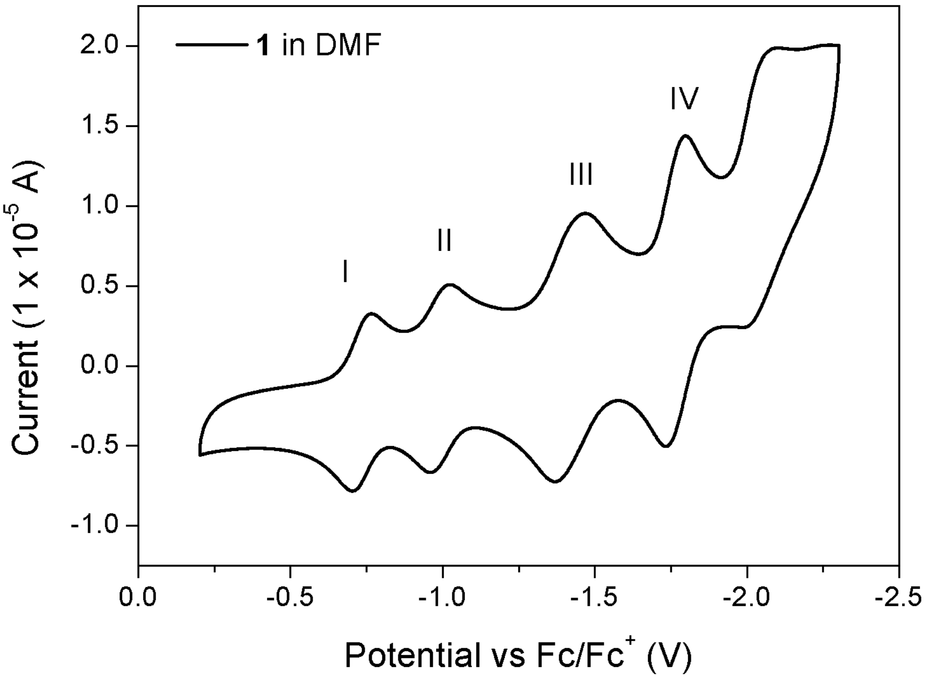

Figure S14. Cyclic voltammogram of **1** (0.5 mM) in DMF, with 0.1M $TBAPF_6$ as electrolyte. Scan rate: 0.1 V/s.

Redox potentials vs Fc/Fc^{+} (V)	I	II	III	IV
E_{red}	-0.764	-1.022	-1.471	-1.798
E_{ox}	-0.702	-0.961	-1.367	-1.734
$E_{1/2}$	**-0.733**	**-0.992**	**-1.419**	**-1.766**

Table S3. Redox potentials of **1** in DMF

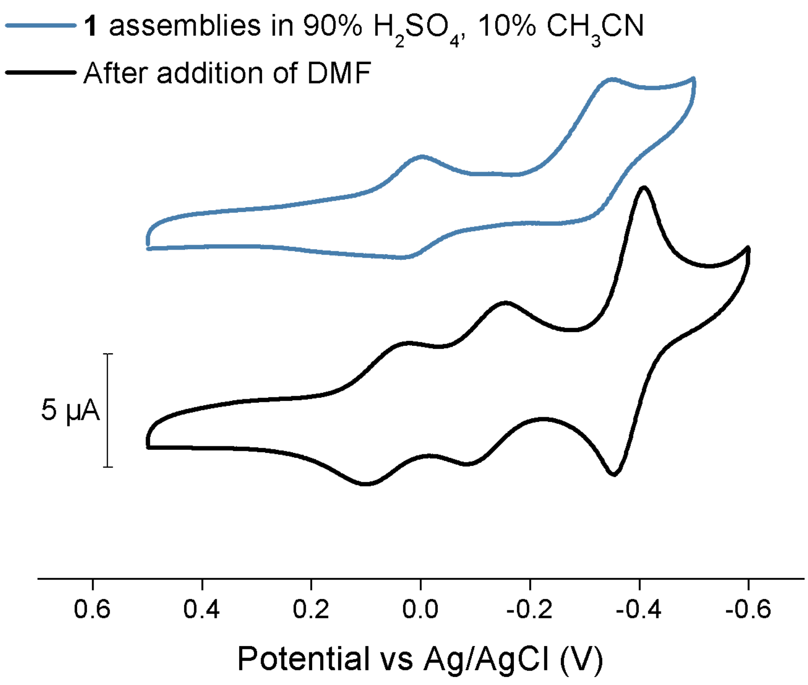

Figure S15. Comparison of the cyclic voltammograms of **1** (1.4 mM) in 90% H_2SO_4 (0.1M) 10% CH_3CN before and after the addition of the same volume of DMF (c_{new} = 0.7 mM). Scan rate: 0.1 V/s.

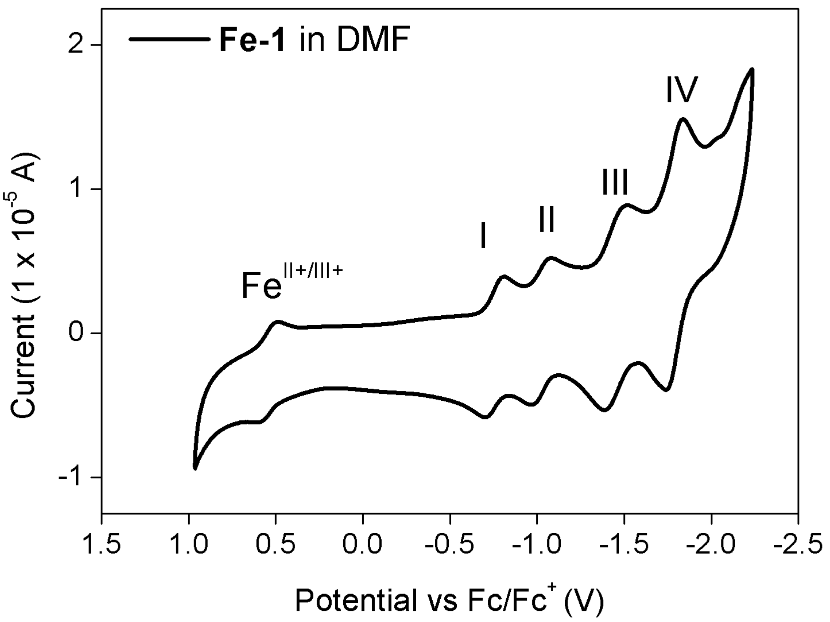

Figure S16. Cyclic voltammogram of **Fe-1** (5.3 mg in 2mL, 0.25 mM) in DMF, with 0.1M $TBAPF_6$ as electrolyte. Scan rate: 0.1 V/s.

Redox potentials vs Fc/Fc^+ (V)	$Fe^{II/III+}$	I	II	III	IV
E_{red}	0.485	-0.811	-1.081	-1.515	-1.839
E_{ox}	0.601	-0.702	-0.966	-1.387	-1.737
$E_{1/2}$	**0.543**	**-0.757**	**-1.024**	**-1.451**	**-1.788**

Table S4. Redox potentials of **Fe-1** in DMF

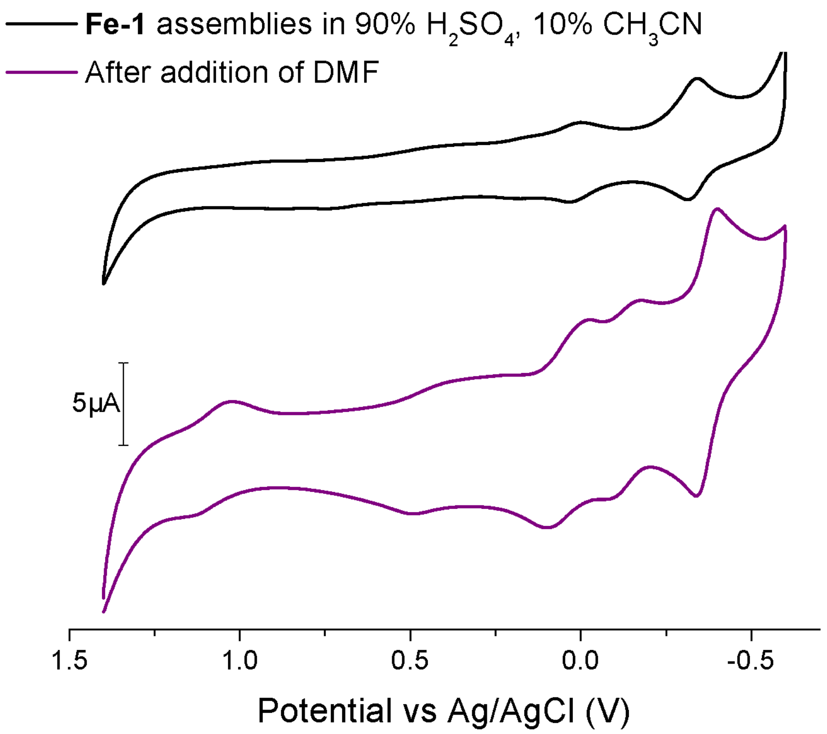

Figure S17. Comparison of the cyclic voltammograms of **Fe-1** (14.8 mg in 2mL, 0.7mM) in 90% H_2SO_4 (0.1 M) 10% CH_3CN and after the addition of the same volume of DMF (c_{new} = 0.35 mM). Scan rate: 0.1 V/s.

DLS & NMR characterisation of assemblies of 1 & Fe-1

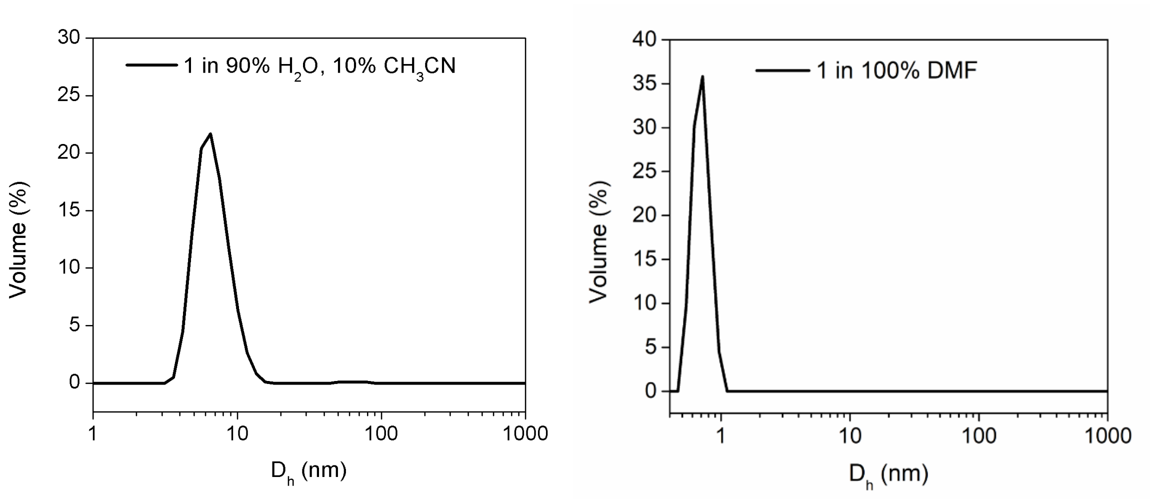

Figure S18. Particle-size distribution curves determined by DLS of: (left) **1** (1.4 mM) in 90% H_2O, 10% CH_3CN, D_h maxima at 6.5 nm; (right) **1** (1.4mM) in 100% DMF, D_h maxima at 0.7 nm (suggesting discrete molecular species only under these conditions).

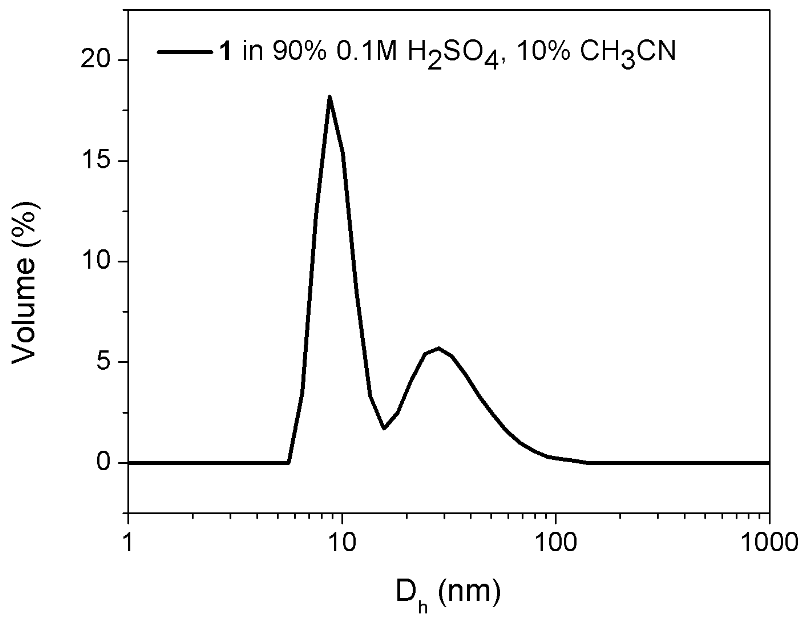

Figure S19. Particle-size distribution curve determined by DLS of **1** (1.4 mM) in 90% H_2SO_4 (0.1M) 10% CH_3CN matching the conditions used in the electrochemical analysis. D_h maxima at 8.7 nm and 28.2 nm. The larger nanostructures are tentatively assigned to assemblies formed through the aggregation of multiple micelles.[3]

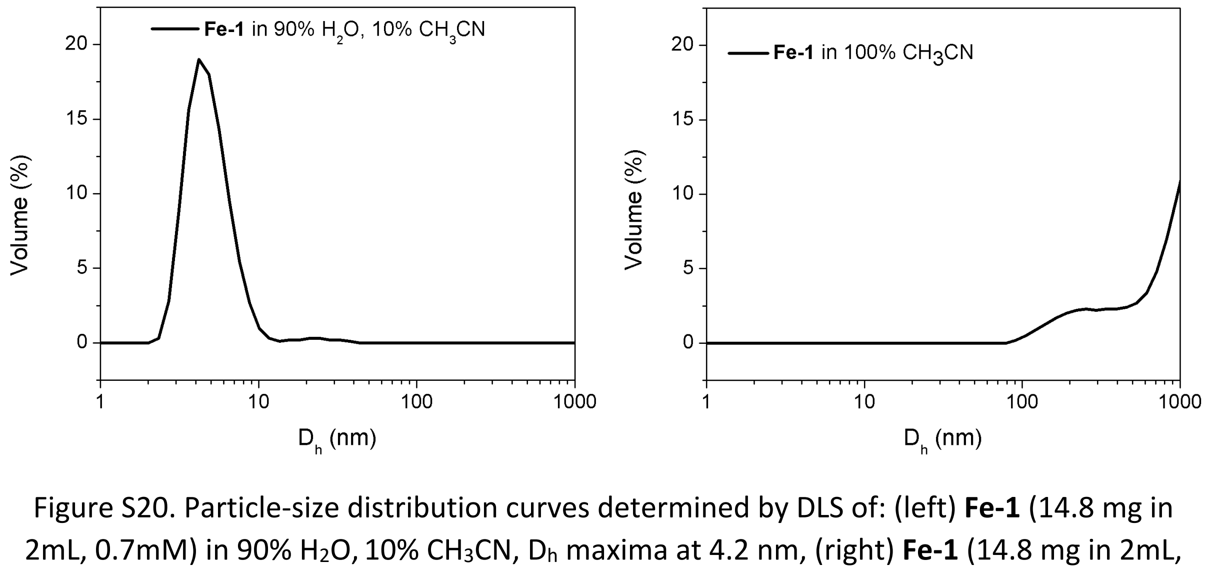

Figure S20. Particle-size distribution curves determined by DLS of: (left) **Fe-1** (14.8 mg in 2mL, 0.7mM) in 90% H_2O, 10% CH_3CN, D_h maxima at 4.2 nm, (right) **Fe-1** (14.8 mg in 2mL, 0.7mM) in 100% CH_3CN (showing loss of the signal for the ca. 4 nm supramolecular assemblies observed in mixed H_2O:CH_3CN solution by DLS (above, left) and TEM (Figure 3b)).

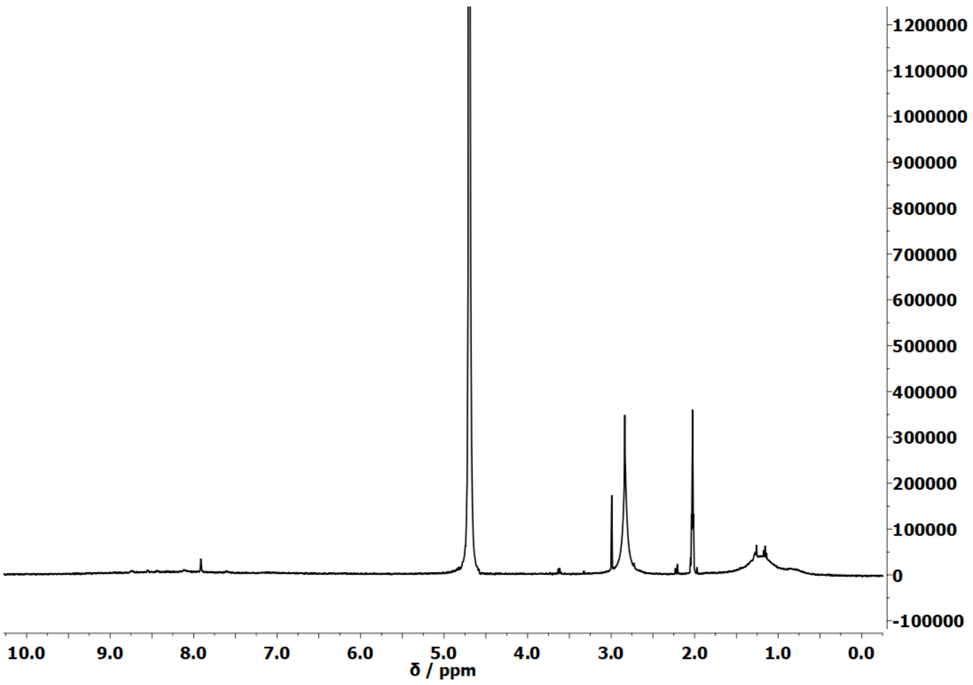

Figure S21. ^{1}H NMR of **1** in 90% D_2O, 10% CD_3CN

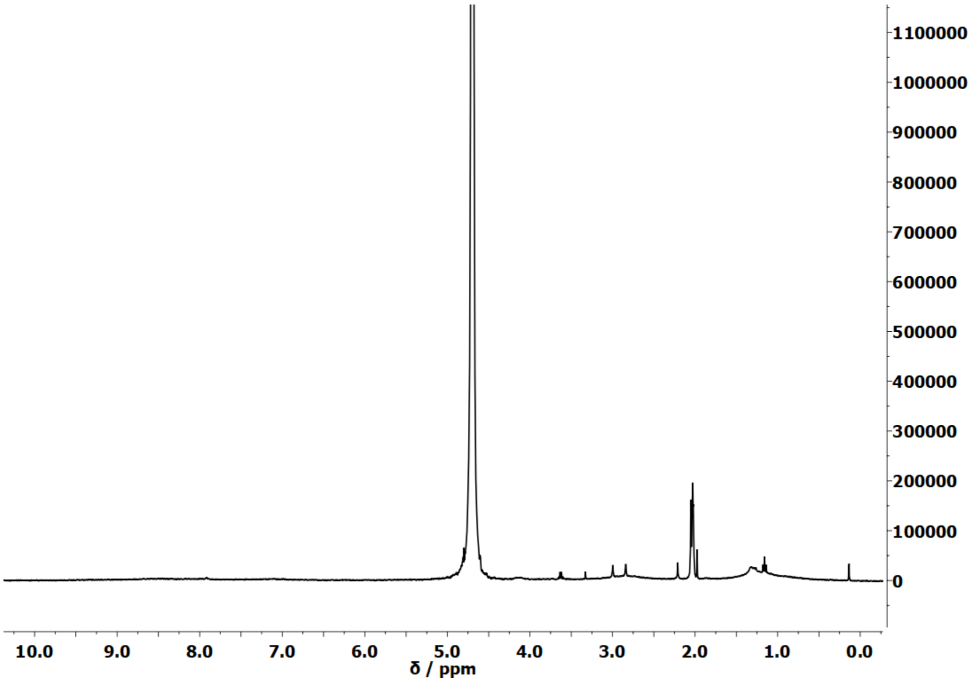

Figure S22. ^{1}H NMR of **Fe-1** in 90% D_2O, 10% CD_3CN

Electrochemistry of 2, 3, and $\{P_2W_{18}\}$, and DLS of 3

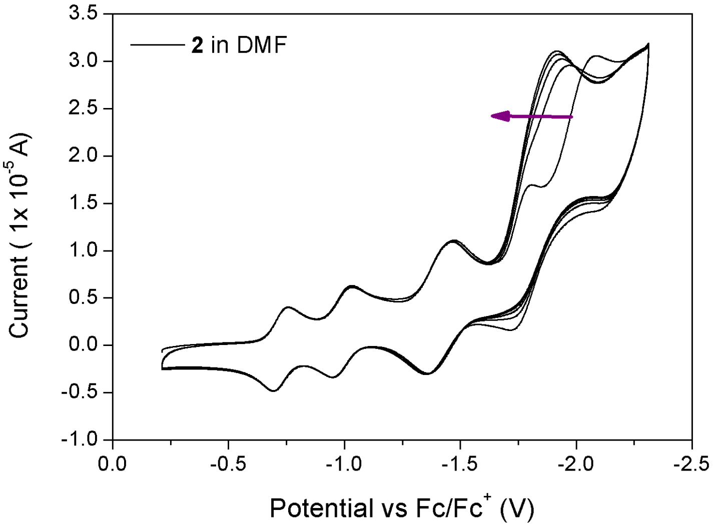

Figure S23. Cyclic voltammogram of **2** (0.5 mM) in DMF, with 0.1M $TBAPF_6$ as electrolyte. Scan rate: 0.1 V/s. Arrow showing direction of peak through progressive cycles as processes IV and V appear to merge, likely a result of TPY reduction)

Redox potential vs Fc/Fc^+ (V)	I	II	III	IV	V
E_{red}	-0.762	-1.031	-1.462	-1.803	-2.057
E_{ox}	-0.694	-0.950	-1.354	1.722	-
$E_{1/2}$	**-0.728**	**-0.991**	**-1.408**	**-1.763**	**-**

Table S5. Redox potentials of **2** in DMF (before the merging of processes IV & V)

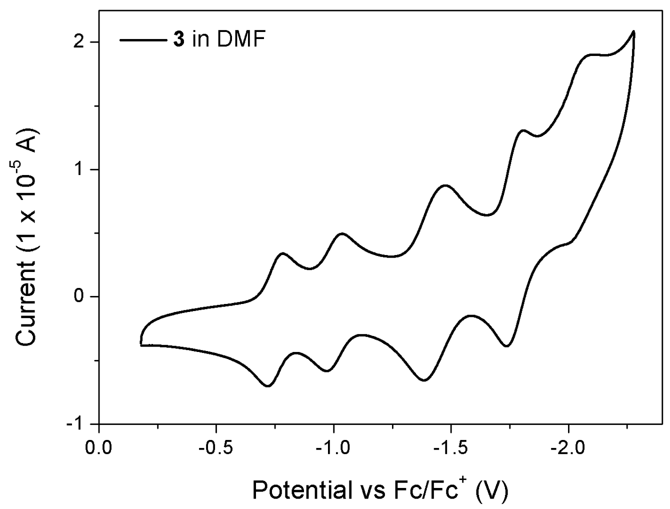

Figure S24. Cyclic voltammogram of **3** (0.5 mM) in DMF, with 0.1M $TBAPF_6$ as electrolyte. Scan rate: 0.1 V/s.

Redox potential vs Fc/Fc^+ (V)	I	II	III	IV
E_{red}	-0.787	-1.040	-1.480	-1.806
E_{ox}	-0.720	-0.976	-1.403	-1.766
$E_{1/2}$	**-0.754**	**-1.008**	**-1.442**	**-1.786**

Table S6. Redox potentials of **3** in DMF

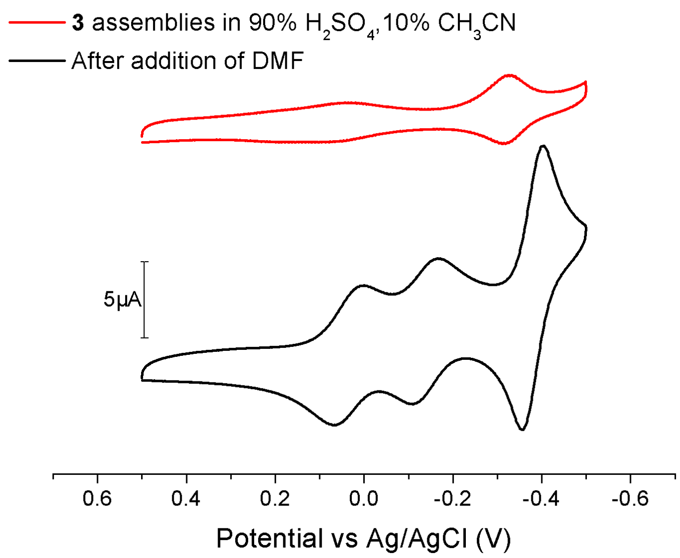

Figure S25. Comparison of the cyclic voltammograms of **3** (1.4 mM) in 90% H_2SO_4 (0.1M) 10% CH_3CN before and after the addition of the same volume of DMF (c_{new} = 0.7 mM). Scan rate: 0.1 V/s.

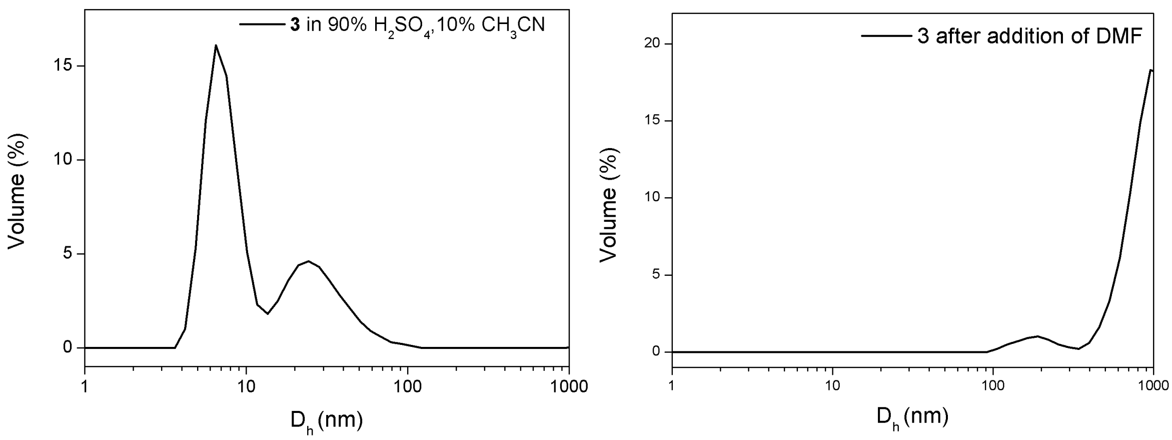

Figure S26. Particle-size distribution curves determined by DLS of: (left) **3** (1.4 mM) in 90% H_2SO_4 (0.1M) 10% CH_3CN matching the conditions used in the electrochemical analysis, D_h maxima at 6.5 nm and 24.4 nm, (right) the solution after the addition of the same volume of DMF (c_{new} = 0.7 mM)

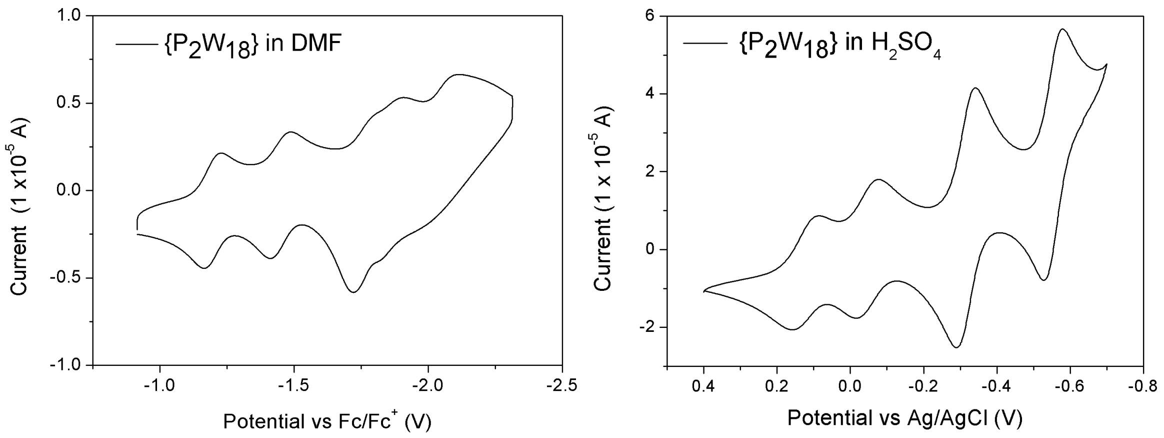

Figure S27. Cyclic voltammograms of: (left) **$K_6[P_2W_{18}O_{62}]$ ({P_2W_{18}}** (0.5 mM) in DMF with 0.1M $TBAPF_6$ as electrolyte, (right) **{P_2W_{18}}** (1.4 mM) in 0.1M H_2SO_4. Scan rates: 0.1 V/s.

References

1. R. Contant, *Inorganic Syntheses*, 1990, **27**, 107.
2. C. R. Graham and R. G. Finke, *Inorg. Chem.*, 2008, **47**, 3679-3686.
3. S. Amin, J. M. Cameron, J. A. Watts, D. A. Walsh, V. Sans and G. N. Newton, *Mol. Syst. Des. Eng.*, 2019, Advance Article. DOI: 10.1039/C9ME00060G
